# High-dimensional single-cell phenotyping reveals extensive haploinsufficiency

**DOI:** 10.1371/journal.pbio.2005130

**Published:** 2018-05-16

**Authors:** Shinsuke Ohnuki, Yoshikazu Ohya

**Affiliations:** 1 Department of Integrated Biosciences, Graduate School of Frontier Sciences, University of Tokyo, Kashiwa, Chiba, Japan; 2 AIST-UTokyo Advanced Operando-Measurement Technology Open Innovation Laboratory (OPERANDO-OIL), National Institute of Advanced Industrial Science and Technology (AIST), Kashiwa, Chiba, Japan; Synthetic and Systems Biology Unit, Hungary

## Abstract

Haploinsufficiency, a dominant phenotype caused by a heterozygous loss-of-function mutation, has been rarely observed. However, high-dimensional single-cell phenotyping of yeast morphological characteristics revealed haploinsufficiency phenotypes for more than half of 1,112 essential genes under optimal growth conditions. Additionally, 40% of the essential genes with no obvious phenotype under optimal growth conditions displayed haploinsufficiency under severe growth conditions. Haploinsufficiency was detected more frequently in essential genes than in nonessential genes. Similar haploinsufficiency phenotypes were observed mostly in mutants with heterozygous deletion of functionally related genes, suggesting that haploinsufficiency phenotypes were caused by functional defects of the genes. A global view of the gene network was presented based on the similarities of the haploinsufficiency phenotypes. Our dataset contains rich information regarding essential gene functions, providing evidence that single-cell phenotyping is a powerful approach, even in the heterozygous condition, for analyzing complex biological systems.

## Author summary

Diploid organisms harboring a wild-type gene and a loss-of-function mutation are called heterozygotes. They are expected to have weak or no individual phenotypes because the mutation is compensated for by the intact allele. The dominant inheritance of phenotypes in heterozygotes is an exceptional phenomenon called haploinsufficiency. Haploinsufficiency was thought to be a rare occurrence; however, a sensitive technique called high-dimensional single-cell phenotyping challenges this perspective. Investigations of single-cell phenotypes revealed that a large extent of the essential genes in yeast exhibit haploinsufficiency. Our analyses also provided crucial information on gene functional networks based on haploinsufficiency phenotypes. This work shows that high-dimensional single-cell phenotyping is a useful tool that can be used to better understand complex biological systems.

## Introduction

The concepts of dominance and recessiveness were originally formulated by Gregor Mendel [1] and are still fundamental to modern genetics. Loss-of-function mutations are mostly recessive and rarely dominant in diploid organisms. Haploinsufficiency is a rare manifestation of the dominant phenotype arising from a copy of a loss-of-function mutation in the heterozygous state and was initially studied in *Drosophila* [2]. There is great interest in haploinsufficient genes because the loss of 1 functional allele is linked to human diseases including cancer and tumorigenesis, developmental and neurological disorders, and mental retardation [3]. Therefore, it is challenging to determine the number of genes in the genome that are sensitive to 1-copy gene loss [4,5].

Two models have been developed to explain the occurrence of haploinsufficiency. As can be seen in dosage-dependent sex determination in *Drosophila* [6], a reduction in the gene copy number affects regulatory genes working at a threshold level. Some proteins are likely produced at the lowest level possible for proper function. Therefore, haploinsufficiency may simply be due to a reduction in protein level in the heterozygous state, which is referred to as the insufficient amount hypothesis. A second theory, referred to as the balance hypothesis, predicts that the stoichiometry of various protein components is important for maintaining the integrity of a protein complex [7]. In yeast, representative haploinsufficient genes include cytoskeletal components such as actin (Act1) [8] and tubulin (Tub1) [9] as well as components of protein complexes such as spindle pole body component (Ndc1) [10] and myosin (Mlc1) [11]. In these circumstances, gene overexpression also results in an imbalance of the components and shows similar phenotypic consequences of 1-copy gene loss.

Genome-wide studies have been performed to investigate haploinsufficient growth phenotypes in the budding yeast *Saccharomyces cerevisiae*. Among 5,900 yeast genes analyzed, approximately 3% (184 mutants) exhibited haploinsufficient growth in rich media [12]. Many of the yeast haploinsufficient genes were functionally related and related to ribosomal function [12], suggesting a significant contribution of ribosomal function to rapid growth. By further investigating the growth phenotypes under limited nutrient conditions, up to 20% of the genome was found to display a haploinsufficient abnormality [13]. A recent systematic screen of another budding yeast, *Candida albicans*, revealed that 10% of the genes in the genome influenced cell size under optimal growth conditions [14]. However, the extent of haploinsufficiency was still restrictive, and little is known about the functional relationships between these genes.

One approach to identify haploinsufficiency is to monitor the phenotypes from different perspectives. Cell morphology is an attractive target for intensive analyses because it reflects a wide variety of cellular events, and hundreds of traits can be analyzed [15]. In this study, we investigated the haploinsufficiency of 1,112 heterozygotes of yeast essential genes using high-dimensional phenotyping with 501 morphological traits. We found that more than half of the essential genes displayed haploinsufficiency under optimal growth conditions, indicative of extensive haploinsufficiency. Similar haploinsufficiency phenotypes were caused by heterozygous deletion of functionally related essential genes. Correlation networks of haploinsufficient genes provided a global view of their functional relationships. Our dataset offers useful resources for the study of essential gene functions in *S*. *cerevisiae*.

## Results

### Frequently observed morphological haploinsufficiency in yeast essential genes

**Fig 1:**
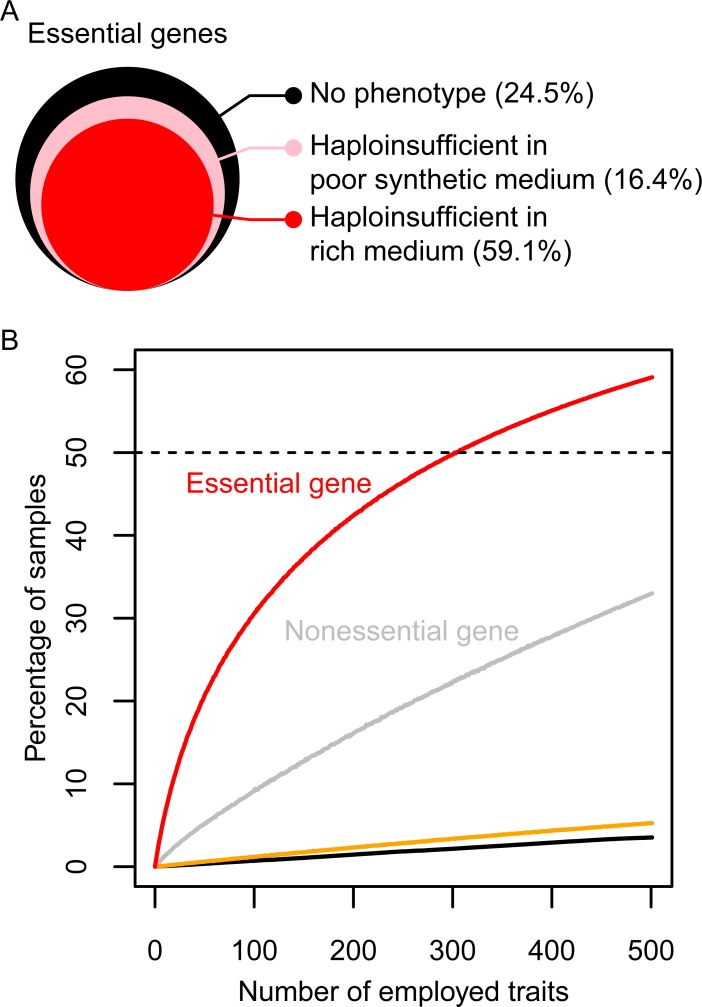
Detection of morphological haploinsufficiency. (A) Graphical representation of heterozygous essential gene-deletion mutants exhibiting haploinsufficiency phenotypes (S1 and S2 Data). The size of the colored area shows the relative ratio of the corresponding heterozygotes. (B) Percentage of heterozygotes exhibiting haploinsufficiency in at least 1 morphological trait (FDR = 0.01; *P* < 7.64 × 10^−5^) using the morphological traits examined. Red and gray lines indicate essential and nonessential genes, respectively. The orange line indicates the percentage of FPs in 114 wild-type replicates. Black solid line indicates percentage of samples detected by chance as estimated using parametric bootstrap resampling. Black dashed line indicates 50%. The morphological traits were ordered on the x-axis by randomizing the 501 traits (3,000 iterations). FDR, false discovery rate; FP, false positive.

We employed yeast heterozygous diploid strains with deletions in each of the essential genes and examined haploinsufficiency in terms of its effects on morphology (morphological haploinsufficiency) by performing single-cell high-dimensional phenotyping. To minimize variation due to inconsistencies in data acquisition, we collected the cultures after growth to a precise point in early log-phase in rich medium, used the automated image processing system CalMorph [15], and analyzed more than 200 cells for each strain. To exclude technical artefacts due to staining procedures and cell segmentation, automatic discriminators and classifiers built into CalMorph made it possible to obtain high-quality multivariate information on single cells [16]. In addition to 220 mean and 61 ratio morphological parameters, 220 variance parameters—which represent variance of the single-cell distribution in morphology—were extracted. To detect phenotypic abnormalities, a generalized linear model (GLM) was applied (S1 Table). As expected, haploinsufficient morphological phenotypes were rarely observed. Of all the combinations between 501 traits and 1,112 heterozygous diploids, only 0.764% (4,258 assays) were significantly different from the wild-type diploid based on a 1-sample 2-sided test (false discovery rate [FDR] = 0.01; *P* < 7.64 × 10^−5^; S1 Fig). However, an analysis of morphological phenotypes in each strain revealed a large number of haploinsufficient genes. A total of 59.1% (657 heterozygous diploids, S2A Table) of the heterozygous deletion mutants exhibited differences compared with the wild-type diploid in at least 1 of the morphological traits examined (FDR = 0.01; *P* < 7.64 × 10^−5^; red area in Fig 1A and S2B Fig). The number of abnormal mutants detected for each trait was relatively small, mostly within the IQR between 2 and 12. We estimated that the rate of false positive (FP) abnormal mutants detected by chance in our analysis was 6% (Fig 1B, black line), which was almost the same as the number of abnormal replicates in the wild type (Fig 1B, orange line). This confirmed that our statistical estimation of the number of haploinsufficiency phenotypes was not overestimated. We used an alternative approach to estimate the number of haploinsufficient mutants following dimensional reduction. A large number of heterozygotes (40% of 1,112) still displayed haploinsufficiency in at least 1 of the 20 principal components (PCs) covering 60% of variance of the morphological phenotypes (FDR = 0.05, S3C Fig).

### Single-cell phenotyping used to detect morphological haploinsufficiency

We found that the cumulative number of haploinsufficient mutants increased with an increase in the number of morphological traits examined (Fig 1B, red line). Mean parameters—and, more effectively, variance parameters—contributed to haploinsufficiency detection (S4A Fig), highlighting the importance of single-cell phenotyping. Ratio parameters were less important because the cumulative number of haploinsufficient mutants reached 98% without the ratio parameters (S4B Fig, light blue line). We next investigated whether the differences between the morphologies of haploinsufficient mutants increased or decreased phenotypic variance and found significantly more phenotypic variance in the 657 haploinsufficient strains than in the other strains (S5 Fig; *P <* 0.01 after Bonferroni correction and Mann–Whitney U test). This observation is consistent with the previous finding that decreasing dosage with the use of conditional alleles often results in increased morphological variation within populations of isogenic cells [17]. Therefore, one widespread function of essential genes is to stabilize morphological phenotypes.

### Comparison between essential and nonessential genes

We counted the frequency of haploinsufficiency in nonessential genes by examining 100 randomly selected heterozygous gene-deletion mutants. For the 501 traits, 33% of the heterozygous diploids showed haploinsufficiency at the same threshold (*P* < 7.64 × 10^−5^; Fig 1B, gray line). Therefore, the frequency of haploinsufficiency in essential genes (Fig 1B, red line) was approximately 2-fold greater than that in nonessential genes (Fig 1B, gray line). We noted previously that 65% of the haploid mutants with nonessential deletions were morphologically distinct [15], indicating that the morphological phenotypes in heterozygous diploids were less commonly observed than those in haploid deletion mutants (S2B Table). These analyses indicated that essential genes have a large impact on haploinsufficient morphological phenotypes.

### Morphological haploinsufficiency in poor synthetic medium

We tested the morphological haploinsufficiency of heterozygous diploids under nutrient-limited growth conditions in 50 randomly selected heterozygous deletion mutants, which exhibited no haploinsufficiency in rich media. After growth in poor synthetic medium, 40% (16.4% out of 40.9%) of heterozygous diploids that exhibited no obvious morphological phenotypes in rich media exhibited haploinsufficiency in at least 1 of the morphological traits (*P* < 7.64 × 10^−5^; Fig 1A, pink area). This indicated that up to 75.5% (59.1% + 16.4%) of the heterozygous diploids exhibited phenotypes either in rich or poor synthetic medium.

### Functional defects associated with morphological haploinsufficiency

**Fig 2:**
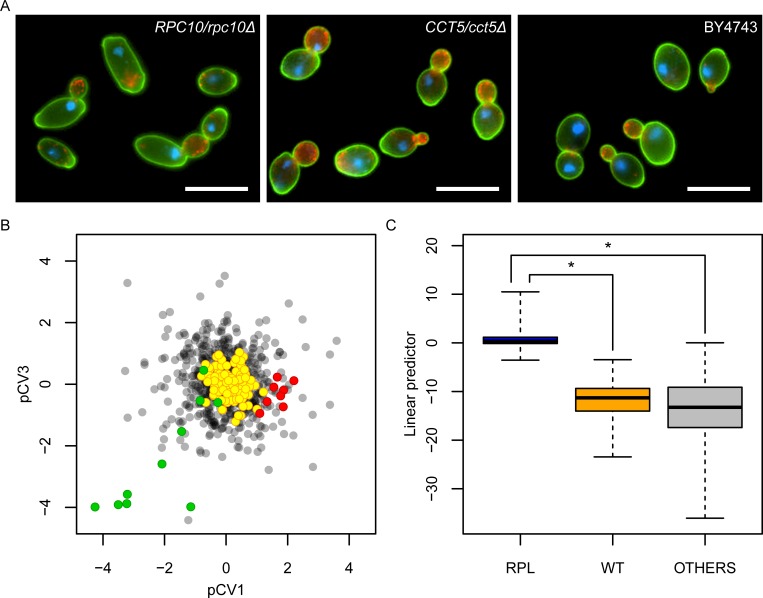
Function-specific haploinsufficiency phenotypes. (A) Images of heterozygous mutants for RNA pol II (*RPC10*/*rpc10*Δ), chaperonin CCT (*CCT5*/*cct5*Δ), and wild type. Cells were stained with FITC-conjugated concanavalin A for cell wall (green), rhodamine-phalloidin for actin (red), and DAPI for nuclear DNA (blue), and presented with pseudo-coloring. Scale bar indicates 10 μm. (B) Biased distribution of heterozygous mutants in phenotypic space. After CCA was performed, scores of CVs of the haploinsufficiency phenotypes were plotted in pCV1/pCV3 orthogonal space. Green and red circles indicate mutants heterozygous for RNA pol II and chaperonin CCT, respectively. Gray and yellow circles indicate the remaining heterozygotes and 114 wild-type replicates, respectively. (C) Linear predictor of logistic regression showing phenotype specificity for the cytosolic large ribosomal subunit (GO:0022625) heterozygous mutants (S3 Data). Linear predictor was calculated by linear combinations of 10 pCVs (pCV1, pCV2, pCV4, pCV5, pCV12, pCV13, pCV17, pCV18, pCV19, and pCV21) selected by a combinational optimization. Asterisk indicates significant difference (*P <* 0.05 after Bonferroni correction by likelihood ratio test). CCA, canonical correlation analysis; CCT, chaperonin containing TCP-1; DAPI, 4’,6-diamidino-2-phenylindole; FITC, fluorescein isothiocyanate; pCV, phenotype canonical variable; RNA pol II, RNA polymerase II core complex; RPL, ribosomal protein of the large subunit; WT, wild type.

We examined the morphological haploinsufficiency to see whether it could be explained by functional defects of the genes. To investigate the relationship between gene function and a particular haploinsufficiency phenotype, we performed dimensional reduction by principal component analysis (PCA) and canonical correlation analysis (CCA) [18], which is used to explore the relationship between 2 multivariate sets of variables. PCA and CCA successfully compressed all combinations of 444 morphological traits and 830 gene ontology (GO) terms into linear combinations of phenotypic (21 phenotype canonical variables [pCVs]) and gene-function features (21 GO term canonical variables [gCVs]) (S6 Fig). In fact, analysis of the canonical correlation coefficient revealed a significant correlation between phenotype (pCVs) and gene function (gCVs) (*P <* 0.05, Bartlett’s chi-squared test). At a given canonical correlation coefficient in each pair of 21 CVs, no FPs were found by chance after 10,000 iterations of the randomization, indicating that randomized phenotypic data yielded no pairs of CVs. The phenotypic space composed of pCVs was suitable for understanding phenotypic features of haploinsufficient mutants with the same functional defects. For example, exploring the phenotypic space of pCV1 and pCV3 revealed that heterozygotes for RNA polymerase II (RNA pol II) core complex (green) and for subunits of the cytosolic chaperonin containing TCP-1 (CCT) complex (red) were plotted in different directions (Fig 2B). This graphically demonstrated that the heterozygous mutations in RNA pol II and chaperonin CCT caused specific morphologies, namely, large/elongated cell shape and large actin region/nonelliptical cell shape, respectively (Fig 2A, S3A Table).

**Fig 3:**
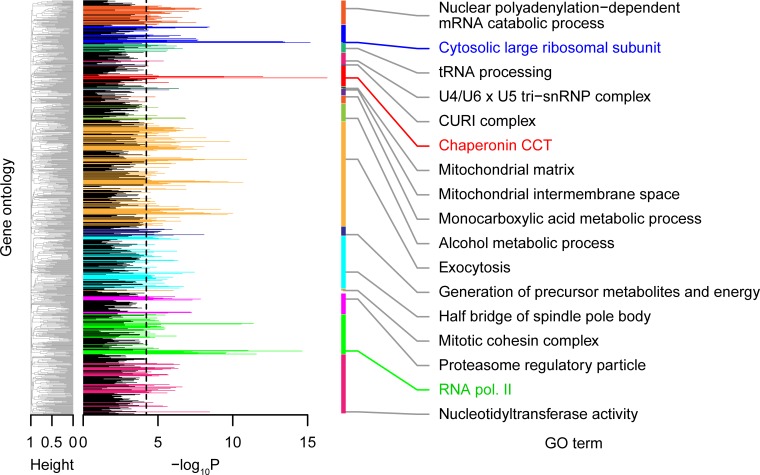
Significant correlation probabilities between gene functions and haploinsufficiency phenotypes. Colored peaks indicate GO terms with significant logistic regression coefficients (*P <* 0.05 after Bonferroni correction, likelihood ratio test) of haploinsufficiency phenotypes. GO terms with minimum *P* values in each GO group are shown on the right side (S3B Table). Cluster dendrograms on the left side were constructed based on the frequency of genes in common between GO terms to classify GO terms into representative groups. Black dashed line indicates threshold of *P <* 0.05 after Bonferroni correction by the likelihood ratio test. CCT, chaperonin containing TCP-1; CURI, CK2-Utp22-Rrp7-Ifh1; GO, gene ontology; RNA pol II, RNA polymerase II core complex; snRNP, small nuclear ribonucleo protein.

The logistic regression analysis can be used to identify the best combinations of pCVs for each GO term, yielding the maximum likelihood prediction of the gene functions with haploinsufficiency phenotypes (e.g., cytosolic large ribosomal subunit [ribosomal protein of the large subunit (RPL)] in Fig 2C). We applied this approach to every GO term and identified 306 GO terms corresponding to 553 genes with a significant correlation between gene function and haploinsufficiency phenotype (*P <* 0.05, likelihood ratio test after Bonferroni correction) (Fig 3 and S3B Table). Therefore, haploinsufficiency phenotypes were associated with gene function in 90% of the haploinsufficient genes, suggesting that the observed phenotypes were mostly explained by functional defects of the genes.

### Correlation analysis of haploinsufficient genes for morphology

To better understand morphological haploinsufficiency, we examined the overlap between haploinsufficient genes for growth [12] and haploinsufficient genes for morphology. A contingency table test showed significant correlations between these 2 datasets (S4 Table; *P <* 0.01 according to Fisher’s exact test), suggesting a common integrant. A previous study revealed that ribosomal function was specifically enriched in haploinsufficiency based on cell growth [12]. Although many ribosomal genes were also morphologically haploinsufficient, specific gene functions were not enriched among the 657 morphologically identified haploinsufficient genes (FDR = 0.1); instead, genes encoding most of the essential cellular processes—such as replication, transcription, translation, protein degradation, membrane trafficking, transporter, cell cycle progression, morphogenesis, and macromolecular synthesis—were represented (S3B Table). We also noted that specific gene functions were not enriched in genes that were not morphologically haploinsufficient (FDR = 0.1). Therefore, careful high-dimensional and single-cell phenotyping detected numerous haploinsufficient genes with functions in diverse cellular processes.

A previous study indicated that genes involved in protein complexes were enriched among haploinsufficient genes related to growth [12]. The genes involved in protein complexes were also significantly enriched among haploinsufficient genes related to morphology (S5 Table, *P <* 0.01 by Fisher’s exact test for 1 side). This suggested that specific gene functions were enriched in both haploinsufficient morphological genes and genes involved in protein complexes. In fact, some gene functions (such as nuclear polyadenylation-dependent mRNA catabolic process, cytosolic large ribosomal subunit, etc.) were enriched with high degrees of protein–protein interaction (S7A Fig, PPI). Similar but distinct gene functions were significantly enriched with high degrees of genetic interaction (S7A Fig; genetic interaction) [19]. By comparing Fig 3 with S7A Fig, a Venn diagram was constructed (S7B Fig), which indicated that among 124 GOs of protein complexes, 70 GOs were enriched in morphologically identified haploinsufficient genes. Therefore, our analysis suggested that numerous haploinsufficient genes are involved in protein complexes with diverse cellular functions.

Haploinsufficient genes are the genes that are sensitive to 1-copy gene loss. Therefore, we next analyzed the correlation of haploinsufficient genes for morphology with overexpression-sensitive genes [20] and with highly expressed genes [21]. We revealed a significant correlation with overexpression-sensitive genes (S8A Fig; Spearman rank correlation coefficient, *P <* 0.01 by *t* test) but failed to detect any correlation with highly expressed genes (S8B Fig, Spearman rank correlation coefficient, *P* = 0.38 by *t* test). However, we detected a significant correlation when we selected genes annotated with a specific GO (S9 Fig, Wald-test, FDR = 0.05). This implies that the correlation between morphologically identified haploinsufficient genes and highly expressed genes is GO specific. Based on these results, we discussed the feasible models for the mechanism of haploinsufficiency (see Discussion).

### Linkage between cell growth and cell morphogenesis

**Fig 4:**
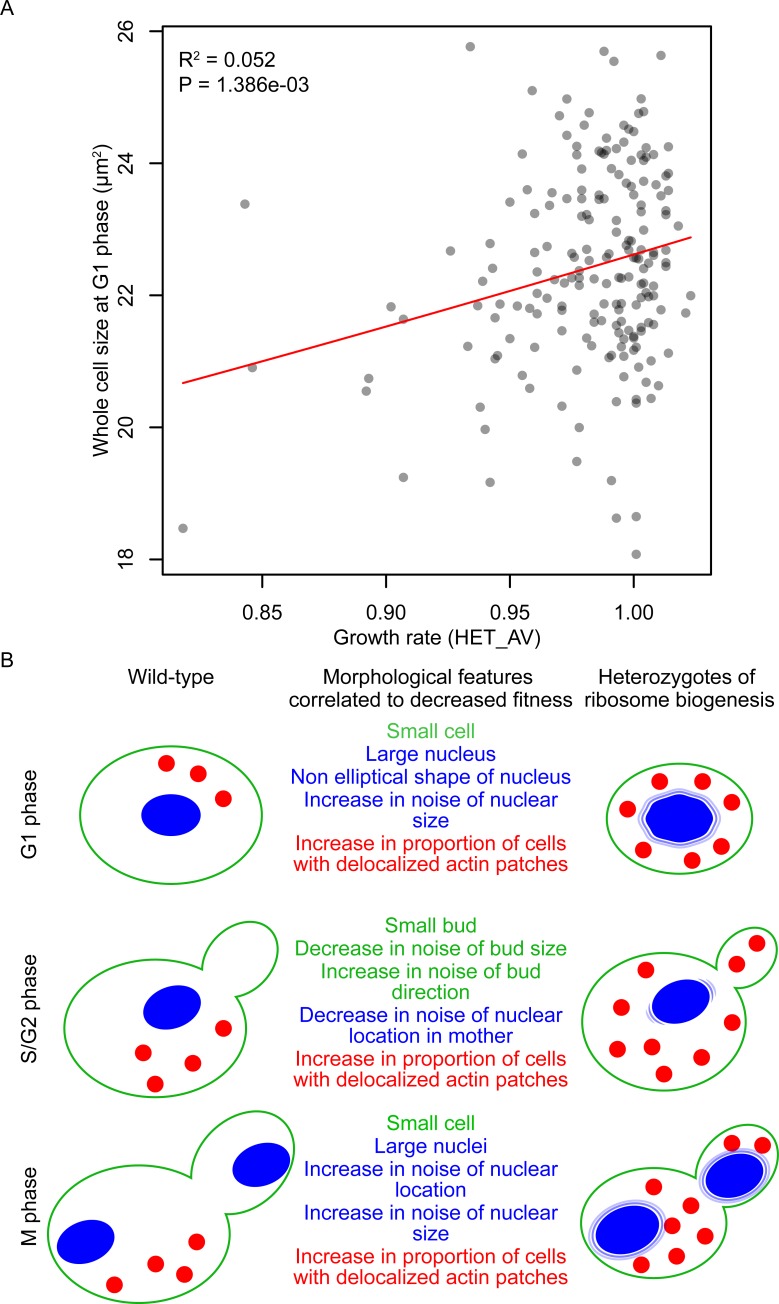
Morphological phenotypes correlated with growth defects in heterozygotes for ribosome biogenesis genes. (A) Distribution of whole-cell size in G1 phase and cell growth. Gray circles indicate 198 heterozygotes for ribosome biogenesis (GO:0042254) genes. x- and y-Axes indicate average growth rate (HET_AV) [12] and average area size of G1 cells (C11-1_A), respectively. Red line indicates linear regression with a gamma distribution. R^2^ indicates coefficient of determination. *P* value was estimated by likelihood ratio test. (B) Illustration of morphological features. Morphological traits correlated to growth (S6 Table) were grouped into 11 features (S7 Table and S10 Fig). Green, blue, and red indicate cell walls, nuclei, and actin patches, respectively.

A previous study of heterozygous diploids showed that the essential genes involved in ribosome biogenesis cause coupling of the growth rate to cell size [22]. Analysis of our dataset confirmed a significant correlation between growth rate and cell size in 198 heterozygous ribosome biogenesis mutants (Fig 4A). Aside from cell size, we revealed that other morphological features were correlated with growth rate in these ribosome biogenesis mutants (S6 Table, likelihood ratio test, FDR = 0.05). Of 163 correlated morphological features, we extracted the independent features (S7 Table and S10 Fig) and summarized them with a schematic representation (Fig 4B). Therefore, our results provide a deeper understanding of a mechanism that may link cell growth with cell morphogenesis, including growth in size, cell cycle progression, actin morphogenesis, and nuclear morphogenesis.

### Correlation of the haploinsufficiency phenotypes

**Fig 5:**
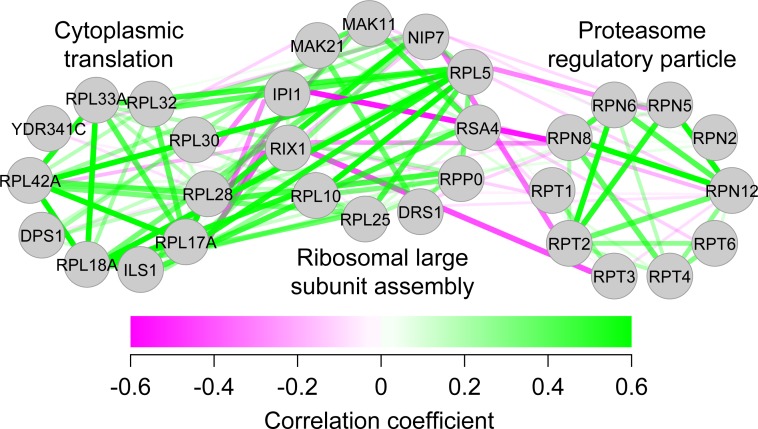
Examples of positive and negative correlations between GO terms. Green and magenta edges indicate positive and negative phenotypic correlations, respectively. Genes related to cytoplasmic translation, ribosomal large subunit assembly, and proteasome regulatory particles were enriched in groups 17, 3, and 47, respectively (S8A Table). GO, gene ontology.

Because the haploinsufficiency phenotypes were due to functional defects of the genes, we further assessed the degree of similarity between the phenotypic profiles of individual haploinsufficient mutants. To do this, a full matrix of gene–gene pairwise similarities was calculated based on the haploinsufficiency phenotypes. Although phenotypic correlation coefficients between all pairs of the heterozygous diploids were distributed largely from –0.23 to +0.23 (mean ± 1 SD), the mean values of those sharing the same GO categories were typically positive (S11 Fig). There were only a few (0.98%) highly correlated (>0.5) cases. We analyzed the interactions with correlations above 0.5 and found many cases of interactions within the protein complex GO (S12 Fig). Therefore, the similar haploinsufficiency phenotypes were associated with the deletion mutants in the same GO categories. After dimensional reduction by CCA, a high level of precision and recall curve for GO terms was achieved (S13 Fig), indicating that the positive correlation coefficient had substantial predictive power for gene function. We compared the precision-recall characteristics of our phenotypic data to the results from other high-throughput studies (S14 Fig) and found that our data (red) were almost as precise and sensitive as protein interaction [23] (green) and microarray co-expression data [24] (purple) and were more predictable than phosphoprotein (orange) [25] and genetic interaction data [26] (blue). We then tested pairs of correlation coefficients between representative functional gene groups (S8A Table) and observed both positive and negative correlations. For example, the mean value between “cytoplasmic translation” and “ribosomal large subunit assembly” (both involved in protein synthesis) was positive, while that between “ribosomal large subunit assembly” and “proteasome regulatory particle” was negative (Fig 5). The negative correlations reflected the opposing nature of the cellular processes, namely, protein synthesis and degradation. Our results strongly suggest that positive and negative correlations of the haploinsufficiency phenotypes reflect functional relationships in cellular processes.

**Fig 6:**
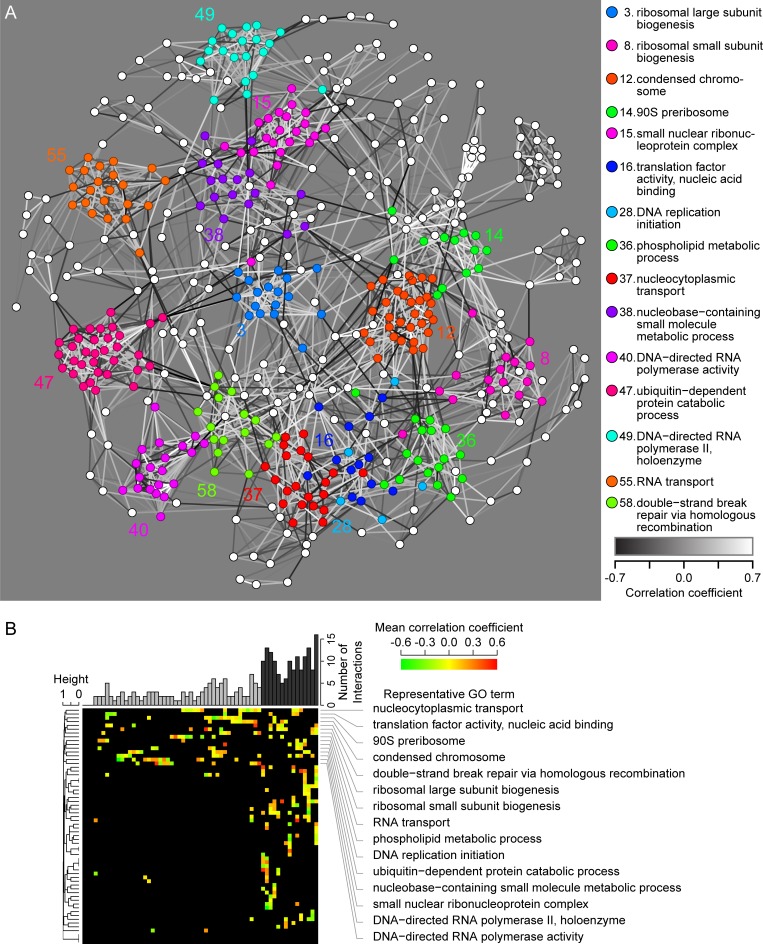
Global view of functional relationships between haploinsufficient genes. (A) Graphical representation of haploinsufficient gene network. White and black edges indicate positive and negative phenotypic correlation. Transparency of edges indicates absolute value of the correlation coefficient. Colored nodes represent 285 haploinsufficient genes belonging to core gene groups. (B) Heat map of the phenotypic correlation coefficient between each pair of functional gene groups. Pairs with significant phenotypic correlation coefficients are indicated with colors in the heat map. The upper bar plot indicates the number of pairs with significant similarity. The 15 identified core functional gene groups (black bars) are shown by the representative GO term on the right side. The dendrogram is constructed based on the proportion of significant correlation. GO, gene ontology.

We used correlations between haploinsufficiency phenotypes to construct global functional maps among the yeast essential genes. Based on the patterns of the relationships, we systematically mapped 513 essential genes belonging to 46 GO terms (Fig 6A and S8B Table). We observed 15 core gene groups containing 285 haploinsufficient genes with functions in DNA replication, transcription, nuclear transport, translation, phospholipid metabolism, and protein degradation that served as a hub: these genes were related directly and/or indirectly to all of the other genes. Pairwise testing did not detect significant phenotypic correlations between the core gene groups (Fig 6B), indicative of the different and diverse functions of the hub genes. These phenotypic relationships provide a global view of the functional relationships between large numbers of haploinsufficient genes.

## Discussion

Comprehensive single-cell phenotyping of heterozygous diploids in budding yeast revealed that more than half of the essential gene mutants are haploinsufficient in morphology. Up to 76% of the heterozygous diploids showed distinct morphological phenotypes either in rich or minimal media. High-dimensional phenotyping with many points of view yielded an even larger number of haploinsufficient mutants. This suggests that future high-dimensional assays will identify more haploinsufficient genes that are linked to human diseases. Among phenotypic values acquired from hundreds of individual cells, the variance value of the traits was found to be more effective than others, demonstrating the importance of single-cell phenotyping. The morphological phenotypes of the haploinsufficient heterozygotes could be mainly explained by gene function. There was morphological similarity within the deletion mutants of functionally related genes, as evidenced by dense gene clusters with rich functional information, and functional networks based on morphological similarity.

Phenotypes can be perturbed by environmental changes, epigenomic effects, and/or experimental artefacts [27]. To demonstrate that the observed haploinsufficiency phenotypes were due to chromosomal heterozygous deletions, we determined whether the haploinsufficiency phenotypes could be explained by gene-functional defects. We found that 90% of genes with functional defects (553 of 610 haploinsufficient genes with reliable GO annotations) were associated with the phenotypes of heterozygous diploids. The strong correlation between gene function and the haploinsufficiency phenotype provides concrete evidence that a decrease in the gene dosage could result in malfunctioning in a large proportion of essential genes.

Given the results from previous comprehensive studies of haploinsufficient genes, it was quite surprising that such a large proportion of essential genes displayed haploinsufficiency. Studies in budding yeast revealed that approximately 9% of essential genes in the genome are haploinsufficient for growth in rich medium [12]. A careful survey of the *Drosophila* genome showed that only 56 loci were associated with an altered phenotype when present as a single copy [28]. Compared with results from a previous study, we found that most of the genes involved in essential cellular processes were haploinsufficient in terms of morphology.

Genes encoding components of protein complexes were significantly enriched among the haploinsufficient genes, which supports the balance hypothesis. In addition, the significant correlation between overexpression-sensitive and haploinsufficient genes supports the balance hypothesis discussed previously [7,12]. On the other hand, many genes encoding noncomplex enzymes were also haploinsufficient, which supports the insufficient amount hypothesis. Although we failed to detect a significant correlation between highly expressed and haploinsufficient genes on the whole, we detected a significant correlation when we selected haploinsufficient genes annotated with specific GO terms, including carbohydrate-derivative biosynthetic process (GO:1901137 in alcohol metabolic process group; S9 Fig), RNA methyl transferase activity (GO: 0008173 in tRNA processing group; S9 Fig), and mitotic cohesin complex (GO: 0030892). The correlation between highly expressed and haploinsufficient genes supports the insufficient amount hypothesis, and haploinsufficiency of these genes can be easily explained by this hypothesis. Therefore, according to our analysis, it is conceivable that both the insufficient amount and balance hypotheses are correct. Further study will be necessary to determine which hypotheses are applicable for each haploinsufficient gene.

Our dataset will provide researchers with a tool for gaining insights into the functions of yeast essential genes. First, haploinsufficiency phenotypes can be used to understand the function of essential genes. Compared with the various pleiotropic phenotypes frequently observed in conditional lethal mutants [29,30], haploinsufficiency phenotyping is equally reliable. Second, phenotypic similarities between heterozygous diploids can be used either to identify previously known functional connections or propose previously unknown functional connections. It should be noted that phenotypic similarities between the nonessential deletion mutants were used to predict gene function [15]. We observed both positive and negative correlations between haploinsufficiency phenotypes, suggesting that high-dimensional single-cell phenotypes reflect functional relationships in the cellular network. Third, it would also be interesting to compare haploinsufficient genes observed under different conditions. Because more than 1,000 chemical genetic assays revealed a growth defect for all deletion mutants [31], phenotyping in multiple environments is a promising strategy. Therefore, as is the case for growth phenotyping [13], morphological phenotyping under different growth conditions will reveal important aspects of gene function. Finally, comparisons between haploinsufficient and chemical-induced morphological profiles [32] will be used to explore intracellular drug targets. We will be able to make more precise predictions by integrating haploinsufficient morphological profiles with chemical-genetic interaction profiles [33] or other gene features. These will give us additional tools for drug target prediction.

## Materials and methods

### Strains and culture conditions

A collection of heterozygous gene-deletion mutants was purchased from EUROSCARF (http://www.euroscarf.de). Essential genes were defined previously [34]. The yeast diploid strain BY4743 was used as the wild type. Strains heterozygous for 1,112 essential genes and 100 randomly selected nonessential genes and the wild-type strain were cultured under optimal growth conditions at 25°C in nutrient-rich yeast extract peptone dextrose (YPD) medium containing 1% (w/v) Bacto yeast extract (BD Biosciences, San Jose, CA), 2% (w/v) Bacto peptone (BD Biosciences), and 2% (w/v) glucose, which was prepared as described previously [15]. Strains heterozygous for 50 essential randomly selected genes and the wild-type strain were cultured under severe growth conditions at 37°C in nutrient-poor synthetic minimal dextrose (SD) medium, which was prepared as described previously [35].

To minimize variation due to inconsistencies in data acquisition, we used a precise protocol to prepare yeast cells growing in early log-phase. Strains were activated from the freezer stock by streaking onto YPD agar plates and incubating for 3 d at 25°C. Three colonies from each strain were inoculated into 2 mL of YPD liquid medium in a 20-mL glass test tube (Iwaki, Shizuoka, Japan), and the liquid culture was incubated on a rotator (30 rpm with RT-50; TITEC, Saitama, Japan) at 25°C for 20 h. Then, the cells were transferred into 20 mL of fresh liquid medium in a 100-mL conical flask (Iwaki). The cells were further incubated in a shaking water bath (110 rpm with LT10-F; TITEC) at 25°C at least for 16 h. A total of 5.0 × 10^6^ cells at log-phase were harvested and used for fixation and fluorescence staining.

### Fluorescence staining, microscopy, and image processing

Yeast cells were fixed for 30 min in growth medium supplemented with formaldehyde (final concentration, 3.7%) and potassium phosphate buffer (100 mM [pH 6.5]) at 25°C as described in [36]. Yeast cells were then collected by centrifugation at room temperature and further incubated in potassium phosphate buffer containing 4% formaldehyde for 45 min. Next, actin staining was performed by overnight treatment with 15 U/mL rhodamine-phalloidin (Invitrogen, Carlsbad, CA) and 1% Triton-X in phosphate-buffered saline (PBS). Staining of cell-surface mannoproteins was performed by 10-min treatment with 1 mg/mL fluorescein isothiocyanate (FITC)-conjugated concanavalin A (Sigma-Aldrich, St. Louis, MO) in P buffer (10 mM sodium phosphate and 150 mM NaCl [pH 7.2]). After washing twice with P buffer, the yeast cells were mixed with mounting buffer (1 mg/mL *p*-phenylenediamine, 25 mM NaOH, 10% PBS, and 90% glycerol) containing 20 mg/mL 4’,6-diamidino-2-phenylindole (DAPI; Sigma-Aldrich) to stain DNA. Finally, the specimens were observed using an Axio Imager microscope equipped with a 6100 ECplan-Neofluar lens (Carl Zeiss, Oberkochen, Germany), a CoolSNAP HQ cooled charged coupled device (CCD) camera (Roper Scientific Photometrics, Tucson, AZ), and AxioVision software (Carl Zeiss).

Image processing was performed using CalMorph (version 1.3) software designed for diploid yeast strains [37]. CalMorph can collect a large amount of data regarding many morphological parameters of individual cells such as cell cycle phase and cell form from a set of photographs of cell walls, nuclei, and actin cytoskeletons. The CalMorph user manual is available at the *Saccharomyces cerevisiae* Morphological Database (SCMD; http://yeast.gi.k.u-tokyo.ac.jp/datamine/) [38]. Descriptions for each trait were presented previously [15].

### Statistical test for identification of haploinsufficiency phenotypes

To assess haploinsufficiency cell morphology phenotypes statistically, we used the GLM as described previously [39] with minor modifications. The haploinsufficiency phenotypes of heterozygotes were detected using the 1-sample 2-sided test with a null distribution estimated from 114 replicated wild-type strains.

The null distribution for each trait was estimated using 1 of 4 probability density functions (PDFs), as described previously [39]. To minimize the effects of confounding factors affecting microscopic output, we applied the linear model using dummy variables (S1 Table, and S1 Text). The maximum likelihood estimation for each PDF was performed using R function “gamlss” contained in the “gamlss” package [40]. The validity of the null distributions estimated by the wild-type phenotype was assessed using the R-squared value of a quantile–quantile plot. A theoretical distribution for each trait was estimated using the “qqplot” function of the default package using random values (*n =* 11,400) generated from the PDF estimated as a null distribution. To calculate the R-squared value, the theoretical distribution was compared to the distribution of the wild type (*n =* 114). The median of R-squared values among 501 traits was 0.966 (IQR 0.964–0.976), indicating that the selected model and its estimated parameters approximated the distributions of the wild type.

*P* values for each mutant were calculated based on the estimated PDF at 2 sides (low and high tails), such that twice the minimum *P* values were used for statistical tests (1-sample 2-sided test). The FDR was estimated using the R function “qvalue” in the “qvalue” package [41]. Similarly, the number of deletion mutants for nonessential genes was estimated based on the 1-sample 2-sided test with 122 replicated wild-type and 4,718 nonessential gene-deletion mutant strains [15].

The number of mutants detected for at least 1 trait was counted for each threshold (S2 Fig). To estimate the number of samples detected by chance for at least 1 trait, we performed parametric bootstrap resampling using PDFs with maximum likelihood estimations. Random values of 114 samples were generated from each PDF for each parameter. The number of trials (*n =* 3,000) with at least 1 falsely detected trait among 501 traits was counted at each threshold and averaged. In S2 Fig, the confidence intervals from the FPs were estimated by assuming binomial distribution.

### CCA

The purpose of this analysis was to reduce the dimensions from 501 traits and identify biologically important morphological features. We used Z values of 501 traits as a morphometric profile and a Boolean matrix of GO terms as a gene function for each heterozygote. First, we obtained Z values using test statistics of the Wald test using the R function “coeftest” in the “lmtest” package [42] and selected 657 heterozygotes (59%) with significant haploinsufficiency phenotypes at an FDR of 0.01 (Fig 1). We further discarded 47 genes that were annotated by GO terms with fewer than 3 genes. We then selected 830 GO terms that annotated more than 2 genes in the remaining 610 haploinsufficient genes and fewer than 200 genes in the genome with no identical sets of annotated genes. Finally, we used Z values of 444 morphological traits calculated from 610 of the 657 heterozygotes (S2B Fig), such that the 444 traits were detected in at least 1 of the 610 heterozygotes.

To reduce dimensionality, we subjected the morphometric profiles to PCA and the first 17, 29, 50, 91, and 130 PCs (phenotype principal components [pPCs]) contributed more than 0.6, 0.7, 0.8, 0.9, and 0.95, respectively, to the cumulative contribution ratio (CCR). Next, to estimate functional relationships among the 610 genes, we used the structure of 830 GO terms. Dimensionality of GO terms can be reduced by PCA on a Boolean matrix (if a gene was annotated by GO, then its value was 1; otherwise, it was 0), as described previously [43]. The 830 GO terms for the 610 genes were then subjected to PCA, and the first 59, 84, 120, 181, and 346 GO term principal components (gPCs) contributed 0.6, 0.7, 0.8, 0.9, and 0.99, respectively, to the CCR indicating that approximately 346 gene functions were related to the 610 genes.

After projection of Z values on pPCs and a zero matrix on gPCs for 114 replicates of the wild type, we applied CCA to the 130 pPCs and the 346 gPCs, for which the CCRs were 0.95 and 0.99, respectively (S6 Fig). Significance of the canonical correlation coefficient was tested at *P <* 0.05 based on Bartlett’s chi-squared test [44] to obtain 21 morphological features (pCVs) and 21 gene function features (gCVs). To characterize each pCV based on morphological features, linear regression analysis was performed based on the Z value of each trait on pCV and detected at *P <* 0.05 after Bonferroni correction using the F test. Morphological features for each pCV are summarized in S3A Table by successive PCA, as described previously [45].

### Logistic regression with combinational optimization

To detect correlation between GO terms and haploinsufficiency phenotypes, we applied multiple logistic regression analysis to each of the 830 GO terms with combinational optimization techniques for pCVs as explanatory variables. Logistic regressions were performed using the R function “brglm” in the “brglm” package, which was designed to determine a solution to the problem of separation [46]. Combinational optimization was performed using the R function step, in the default package after adaptation of the “brglm” function. A best linear model consisting of 1 of 21 pCVs as an explanatory variable was selected by optimization of algorithms based on Akaike’s Information Criterion (AIC) [47]. The selected model was tested at *P <* 0.05 after Bonferroni correction by the likelihood ratio test using the R function “lrtest” in the “lmtest” package [42].

### Hierarchical cluster analysis for 830 GO terms

The hierarchical cluster analysis (HCA) in Fig 3 was performed using the R function “hca.” Dissimilarity was calculated based on the ratio of shared genes to the union of genes annotated with 2 arbitrary genome-wide GO terms. The GO terms were divided into 20 groups as listed in S3B Table at a height value less than 0.99, such that height was the minimum ratio of the different genes between clusters (complete linkage).

### Precision recall analysis

Precision and recall were calculated as described in Baryshnikova and colleagues (2010) [48] with minor modifications. Correlation coefficients of all (185,745) pairs of 610 genes were calculated using 130 pPC scores or 21 pCV scores. The gene pairs were sorted in ascending order of correlation coefficient and were ranked by the correlation coefficient. The number of gene pairs for which 2 genes were co-annotated by at least 1 of the 830 GO terms listed in S3B Table were counted as true positives (TPs) for each *n*th (*n =* 1, 2, …. 185,745) rank of gene pairs from the first to *n*th rank of the gene pairs. TP was used for the recall. The precision of each TP was calculated by dividing TP by each rank of pairs.

### Pairwise CCA

We first divided the genes into functional gene groups with no common term. The 553 haploinsufficient genes with significant high probabilities of correlation to the gene functions were classified into disjunctional functional gene groups using GO annotations in common. The binary distance between each pair of genes was calculated based on a Boolean matrix of the selected 306 GO terms and used for clustering by the complete linkage method using static branch cutting with a height value less than 1; 62 gene groups were identified, each of which contained from 1 to 33 genes (Fig 6B). To assign the most appropriate GO terms to each gene group, enrichment of GO terms was analyzed using Fisher’s exact test (*P <* 0.05 after Bonferroni correction; S8A Table). In 49 of the groups, more than 1 GO term was enriched. The remaining groups were therefore identified as functional gene groups with no GO terms in common.

Next, we calculated pairwise correlation coefficients between the functional gene groups. To detect significant relationships between the gene groups, we performed pairwise CCA between arbitrary pairs of the 62 gene groups (_62_C_2_ = 1,891) using 21 pCV scores. To eliminate possible detection bias, we used a smaller number of genes than the number of pCVs by reducing dimensionality of genes after applying PCA to the data of heterozygous genes. For pairwise CCA, we applied CCA to pCV scores using the genes and/or the selected PCs as variables, and extracted heterozygote canonical variables (hCVs) as independent components that correlated between the gene groups. We then tested the significance of the canonical correlation coefficient of the first hCV at *P <* 0.05 after Bonferroni correction using Bartlett’s chi-squared test [44]. Among 1,891 pairs of the 62 gene groups, 136 pairs were detected with significant relationships between the gene groups (Fig 6B).

### Networking with phenotypic correlation

A good way to show a global view of functional relationships based on phenotypic correlation is through graphical representation of gene networks. Similarity of phenotypes between the pairs of 513 heterozygotes was calculated using 21 pCV scores and expressed as a correlation coefficient. To visualize the network of the 46 GO term-enriched gene groups (513 genes, S8B Table) with significant relationships to other groups (Fig 6B), we used the R function “qgraph” [49], with which a correlation matrix can be represented as a network. We fed the matrix of the pCV-score–based correlation coefficient after zero filling cells into the “qgraph” of R function when at least 1 of 2 genes in the combination was not significantly related to the first hCV at *P <* 0.05 by *t* test for correlation coefficient (see Pairwise CCA section).

## Supporting information

S1 Text Supporting methods. (DOCX)

S1 Table Model selection and AIC for 501 morphological traits. (A) 440 morphological traits of a continuous value. (B) Sixty-one morphological traits of a discrete value.
(XLSX)

S2 Table List of mutants with abnormal morphologies. (A) Essential genes of heterozygotes with morphological haploinsufficiency phenotypes. (B) Nonessential genes of deletion mutants with abnormal morphological phenotypes.
(XLSX)

S3 Table Correlations of morphological features and gene functions to pCVs. (A) Summary of morphological features correlated with each pCV. (B) List of 830 GO terms and *P* values calculated using multiple logistic regression analysis. GO, gene ontology; pCV, phenotype canonical variable.
(XLSX)

S4 Table Contingency table of haploinsufficiency in essential genes. The set of essential genes of haploinsufficient strains detected in this study was compared to those detected by Deutschbauer et al. [12].
(XLSX)

S5 Table Contingency table of morphological haploinsufficiency and protein complex genes among essential genes. The morphological haploinsufficient genes were compared to those defined as the subunits of protein complexes by Pu et al. [50].
(XLSX)

S6 Table List of morphological traits correlated with growth in heterozygotes for ribosome biogenesis genes. Morphological traits that were significantly correlated with growth were detected at FDR = 0.05 using the likelihood ratio test for a simple regression of the GLM with the PDFs listed in S1 Table. GLM, generalized linear model.
(XLSX)

S7 Table Independent morphological features correlated with growth defects in heterozygotes of ribosome biogenesis genes. Morphological features of the 163 traits listed in S6 Table were summarized through successive PCA, as described previously [45].
(XLSX)

S8 Table Gene functions of haploinsufficient genes. (A) GO terms enriched in each gene group. (B) Genes and annotated GOs in each gene group. GO, gene ontology.
(XLSX)

S1 Fig Detection of the haploinsufficiency phenotypes. Red, gray, and orange bars indicate frequencies of observed haploinsufficiency phenotypes in essential genes (*n =* 557,112), nonessential genes (*n =* 50,100), and wild type (*n =* 57,114), respectively (S1 Data). Family-wise error rate of *P <* 0.05 was estimated by Bonferroni correction (*n =* 557,112).
(PDF)

S2 Fig Detection of the heterozygotes with haploinsufficiency phenotypes. Fraction of the samples in which at least 1 trait was detected at (A) FDR = 0.05 (*P <* 7.57 × 10^−4^), (B) FDR = 0.01 (*P <* 7.64 × 10^−5^), (C) FDR = 0.005 (*P <* 3.02 × 10^−5^), and (D) *P <* 0.05 after Bonferroni correction (*P <* 0.05/557,112) (S1 and S4 Data). False positive indicates the percentage of samples detected by chance, which was estimated for wild type using parametric bootstrap resampling. Error bars indicate 95% CIs. FDR, false discovery rate.
(PDF)

S3 Fig Detection of haploinsufficiency after PCA. (A) CCR (S5 Data). The black bars (left axis) indicate the contribution ratio, the red circles (right axis) indicate the CCR, and the horizontal dashed lines (right axis) indicate 60% of the CCR. The first 20 PCs that covered 60% of variance were used for detection of the haploinsufficiency phenotype. (B) Percentage of detected phenotypes in all tested assays (S5 Data). Red, gray, and orange boxes indicate essential genes (22,240 assays), nonessential genes (2,000 assays), and wild type (2,280 assays), respectively. Number of heterozygotes detected in at least 1 PC at (C) FDR = 0.05 (*P* < 2.38 × 10^−3^), (D) FDR = 0.01 (*P* < 2.12 × 10^−4^), (E) FDR = 0.005 (*P* < 8.19 × 10^−5^), and (F) *P <* 0.05 after Bonferroni correction (*P <* 0.05/22,240) by 1-sample 2-tailed test with Gaussian distribution (S5 Data). CCR, cumulative contribution ratio; PC, principal component; PCA, principal component analysis.
(PDF)

S4 Fig Contribution of single-cell phenotype to detect haploinsufficiency. (A) Comparison among mean, noise, and ratio traits (S6 Data). The percentage of heterozygotes (solid line) and wild type (dashed line) detected in at least 1 trait in 1,112 essential genes were compared among 220 noise traits (green), 220 mean traits (blue), 61 ratio traits (black), and all 501 traits (red). Horizontal lines indicate the maximum percentage with each type of trait. (B) Contribution of “noise + mean” traits (S6 Data). Cyan lines indicate 440 “noise + mean” traits. The other symbols are the same as in panel A.
(PDF)

S5 Fig Increased morphological variation within populations of isogenic cells in haploinsufficient mutants. The phenotypic variance in morphology was calculated in terms of the phenotypic potential (x-axis) (S7 Data), as described previously [37]. Asterisks indicate that applying Bonferroni correction to the Mann–Whitney U test yielded *P <* 0.01.
(PDF)

S6 Fig CCA used for extraction of 21 pairs of CVs. The eye diagram [51] illustrates the CCA procedure. Magenta, red, orange, cyan, green, and blue circles indicate 444 traits, 130 pPCs, 21 pCVs, 21 gCVs, 346 gPCs, and 830 GO terms, respectively. Edges were drawn to have high loadings by cutting with threshold at *P <* 0.05 by *t* test for the loading such that each node has more than 1 relationship to other nodes. CCA, canonical correlation analysis; CV, canonical variable.
(PDF)

S7 Fig Enrichment of GOs with high degrees of PPI and GI. (A) Detection of enriched genes in 2 datasets. In each GO, logistic regression of the interaction degree was applied to the GO annotation of 1,044 genes for PPI [52] and 940 genes for GI [26]. Enrichment of the genes annotated to each GO with a high degree of interaction was assessed using a 1-tailed Wald test for the slope of the linear model at *P <* 0.05 after Bonferroni correction. Color peaks indicate *P* values of the 1-tailed Wald test for each of 830 GOs. The vertical dashed line indicates *P <* 0.05 after Bonferroni correction. Colors of peaks and text for 124 and 201 GOs in PPI and GI, respectively, indicate the GO group and representative GO term, which are the same as in Fig 3. Black peaks and grey texts indicate no correlation was detected at *P <* 0.05 after Bonferroni correction. (B) Venn diagram of the enriched GO terms in the 3 datasets. The GOs detected in each dataset (306 GOs for cell morphology shown in Fig 3, 124 GOs for protein interaction [52], and 201 GOs for genetic interaction [26]) were summarized in a Venn diagram. GI, genetic interaction; PPI, protein–protein interaction.
(PDF)

S8 Fig Correlation between expression level and morphological abnormality. (A) Distribution of copy number limit and morphological abnormality. Gray circles indicate 1,040 essential genes available in both datasets. y- and x-Axes indicate the average copy number limit [20] and morphological abnormality, which was calculated as the Euclidean distance from the mean of the wild type to each heterozygote to obtain a Z value, as described previously [53]. (B) Distribution of protein abundance and morphological abnormality. Gray circles indicate 780 essential genes available in both datasets. y- and x-Axes indicate protein abundance in the cell [21] and morphological abnormality, respectively, which is the same as in panel A. Red line indicates linear regression with a gamma distribution. R^2^ indicates coefficient of determination. *P* values were estimated by likelihood ratio test.
(PDF)

S9 Fig Detection of correlations between protein abundance and function-specific morphological phenotype among selected genes annotated to specific GO terms. Correlation between protein abundance and function-specific morphological phenotype were assessed using linear regression with a gamma distribution. Function-specific morphological phenotypes were identified using the best combination of pCVs for each GO term, as described in Fig 3. Color peaks indicate *P* values of 1-tailed Wald test for the slope of the linear model in each of the 306 GOs described in Fig 3. The vertical dashed line indicates FDR = 0.05. Colors of peaks and text indicate GO group and representative GO term, which are the same as in Fig 3. Black peaks and grey texts indicate that no correlation was detected at FDR = 0.05.
(PDF)

S10 Fig Significant correlations between independent representative morphological traits and growth rate in 198 heterozygous ribosome biogenesis mutants. Each independent morphological feature, as defined by PCs (S7 Table), was represented by the morphological traits with significant PC loading at *P <* 0.05 after Bonferroni correction (*t* test). The 11 PCs reached 60% of CCR. Red lines indicate linear regressions with the PDFs defined in S1 Table. Legends are the same as in Fig 4. R^2^ indicates coefficient of determination. *P* values were estimated by likelihood ratio test.
(PDF)

S11 Fig Density plot of correlation coefficients. The black curve indicates the distribution of morphology correlation coefficients between pairs of 610 haploinsufficient mutants. The red curve indicates the distribution of mean values of the morphology correlation coefficient within the same GO terms.
(PDF)

S12 Fig Representative interaction networks of GOs with high correlation coefficients. The distribution on the left panel shows the means and SDs of the correlation coefficients of the 306 GOs detected in Fig 3. The red circles indicate GOs that are annotated as protein complexes in the CYC2008 database (http://wodaklab.org/cyc2008/) [50]. The vertical dashed line indicates half of the mean of the correlation coefficient. The network graphs on the right panel show representatives of the GOs. The grey nodes indicate essential genes annotated by the representative GOs: cytosolic small ribosomal subunit (GO:0022627), RNA polymerase II (GO:0005665), cytosolic large ribosomal subunit (GO:0022625), SLIK complex (GO:0046695), chaperonin CCT (GO:0005832), RNA polymerase I activity (GO:0001054), tRNA-intron endonuclease complex (GO:0000214), proteasome regulatory particle, lid subcomplex (GO:0008541), and eukaryotic translation initiation factor 2B complex (GO:0005851). The green and magenta edges in each network indicate positive and negative phenotypic correlations, respectively. GO, gene ontology.
(PDF)

S13 Fig Precision versus recall for positive correlation coefficients. TP and FP indicate the numbers of true positives and false positives, respectively. Black and red lines indicate precision/recall values calculated from pPCs (before CCA) and pCVs (after CCA), respectively.
(PDF)

S14 Fig Comparison of precision/recall analysis with other large-scale data. Red: morphological similarity based on haploinsufficient phenotypes (Fig 6A); green: affinity precipitation [23]; purple: similarity of gene expression [24]; blue: synthetic lethality [26]; and orange: phosphorylome network [25].
(PDF)

S1 Data *P* values of 1,112 heterozygotes for essential genes, 100 heterozygotes for nonessential genes, and 114 replicates of wild type after cultivation in YPD. (XLSX)

S2 Data *P* values of 50 heterozygotes for essential genes and 50 replicates of wild type after cultivated in SD. (XLSX)

S3 Data Linear predictor of cell morphology for large ribosomal subunit. (XLSX)

S4 Data Parametric bootstrap of 114 replicates at 4 thresholds. (XLSX)

S5 Data CCR and *P* values after PCA for 114 replicates of wild type and heterozygotes of 1,112 essential genes and 100 nonessential genes. (XLSX)

S6 Data Proportion of detected mutants in each number of traits selected after randomization. (XLSX)

S7 Data Phenotypic potentials of 1,112 heterozygotes for essential genes and 114 replicates of wild type. (XLSX)

## Abbreviations

AIC: Akaike’s Information Criterion
CCA: canonical correlation analysis
CCD: charged coupled device
CCR: cumulative contribution ratio
CCT: chaperonin containing TCP-1
CURI: CK2-Utp22-Rrp7-Ifh1
CV: canonical variable
DAPI: 4’,6-diamidino-2-phenylindole
FDR: false discovery rate
FITC: fluorescein isothiocyanate
FP: false positive
gCV: GO term canonical variable
GLM: generalized linear model
GO: gene ontology
gPC: GO term principal component
HCA: hierarchical cluster analysis
hCV: heterozygote canonical variable
PBS: phosphate-buffered saline
PC: principal component
PCA: principal component analysis
pCV: phenotype canonical variable
PDF: probability density function
pPC: phenotype principal component
RNA pol II: RNA polymerase II
RPL: ribosomal protein of the large subunit
rpm: revolutions per minute
SCMD: *Saccharomyces cerevisiae* Morphological Database
SD medium: synthetic minimal dextrose medium
TP: true positive
YPD medium: yeast extract peptone dextrose medium
