# Supplementary material for: High-dimensional single-cell phenotyping reveals extensive haploinsufficiency
Source: PLoS Biol. 2018 May 16;16(5):e2005130. doi: 10.1371/journal.pbio.2005130 (PMC5955526; doi:10.1371/journal.pbio.2005130)
Supplement: S1 Text — (DOCX) [file pbio.2005130.s001.docx]

Supporting text

# Supporting Methods

## Calculation of noise phenotype

Of the 501 morphological traits computed by CalMorph, 220 traits were coefficients of variation (CV), which provide solid estimates of cell-to-cell heterogeneity. In the CV traits, non-linear dependency on mean trait values was observed, and could be uncoupled by a lowess regression method [1]. This was done using the lowess() function of R with a smoother span of 0.2. We then defined ‘noise traits’ as the residuals (*i.e.*, observed - predicted values) of the model as described previously [2].

## Model selection of probability distributions for the morphological traits

To evaluate the effects of heterozygous deletion of essential genes on cell morphology, an abnormality of the morphological phenotype in each heterozygote was estimated by the probability distribution of wild type in each morphological trait. To this end, we selected four models of a probability distribution suitable to estimate a distribution of wild type in each trait.

1. *Gamma distribution*
   Of the 501 traits, 183 were non-negative values (*e.g.*, length, area size, intensity) and assumed to be gamma-distributed because a special case of the gamma distribution is a Chi-squared distribution, which is known as a distribution of the sum of squares [3-5]. The probability density function (PDF) of the gamma distribution *GA* used in this study is defined below:

$$GA\left( y | \mu,\phi\right)=\frac{y^{(1/\phi^{2}-1)}\exp\left[ -y/\left( \phi^{2}\mu\right) \right]}{\left( \phi^{2}\mu\right)^{\left( 1/\phi^{2} \right)}\Gamma\left( 1/\phi^{2} \right)}$$

where *y* (> 0), *μ* (> 0), and *ϕ* (> 0) are measured value, mean, and dispersion, respectively [6].
2. *Beta distribution*
   Of the 501 traits, 37 ranging from zero to one (*e.g.*, ratio of length, ratio of area) were assumed to be beta-distributed, which was applied to model the behavior of ratios (*e.g.*, ratio of cell size, allele frequencies) [7-9]. The PDF of the beta distribution *BE* used in this study is defined below:

$$BE\left( y | \mu, \phi\right)=\frac{\Gamma\left[ \left( 1-\phi^{2} \right)/{\phi^{2}} \right]y^{{\mu\left( 1-\phi^{2} \right)}/{\phi^{2}}-1}\left( 1-y \right)^{{\left( 1-\mu\right)\left( 1-\phi^{2} \right)}/{\phi^{2}}-1}}{\Gamma\left[ {\mu\left( 1-\phi^{2} \right)}/{\phi^{2}} \right]\Gamma\left[ {\left( 1-\mu\right)\left( 1-\phi^{2} \right)}/{\phi^{2}} \right]}$$

where *y* (0 < y < 1), *μ* (0 < *μ* < 1), and *ϕ* (0 < *ϕ* < 1) are measured value, mean, and dispersion, respectively [6].
3. *Gaussian distribution*
   Of the 501 traits, 220 ranging from negative to positive values were assumed to be Gaussian-distributed, where these traits were normalized to noise phenotypes as described in the “Calculation of noise phenotype” section. The PDF of the Gaussian distribution *NO* is defined below:

$$NO\left( y | \mu, \phi\right)=\frac{1}{\sqrt{2\pi}\phi}\exp\left[ -\frac{1}{2}\left( \frac{y-\mu}{\phi} \right)^{2} \right]$$

where *y* (-∞ < *y* < ∞), *μ* (-∞ < *μ* < ∞), and *ϕ* (> 0) are measured value, mean, and variance, respectively [6].
4. *Beta-binomial distribution*
   Of the 501 traits, 61 traits which were ratios of cells in the specimen were assumed to be beta-binomial distributed, which was applied to model the behavior of the number of successes in the total number of assessments [10-12]. The PDF of the beta-binomial distribution *BB* is defined below:

$$BB\left( y | \mu, \phi\right)=\frac{\Gamma\left( n+1 \right)}{\Gamma\left( y+1 \right)\Gamma\left( n-y+1 \right)}\frac{\Gamma\left( 1/\phi\right)\Gamma\left( y+\mu/\phi\right)\Gamma\left[ n+\left( 1-\mu\right)/\phi-y \right]}{\Gamma\left( n+1/\phi\right)\Gamma\left( \mu/\phi\right)\Gamma\left[ \left( 1-\mu\right)/\phi\right]}$$

where *y* (0, 1, 2, … *n*), *n* (≥ 0), *μ* (0 < *μ* < 1), and *ϕ* (> 0) are the number of particular cells, number of all cells, mean, and dispersion, respectively [6]. We used binomial distribution *BI,* which is a special case of *BB* (*ϕ* = 0), if phi is small enough (i.e*.*, *ϕ* < 10^-10^) or if the Akaike Information Criterion (AIC) of *BI* was lower than that of *BB* after maximum likelihood estimation (MLE).

The PDFs assumed for each trait are listed in S1 Table.

## Generalized linear model (GLM) for confounding factors

Cell morphology abnormalities in heterozygotes were examined by comparing with corresponding wild-type distributions for each trait. The probability distribution of the wild type was performed by fitting the defined PDF (S1 Table) to 114 replicated values of wild type with the MLE in each trait. However, despite the fact that the genotypes were the same, distributions of wild type (n = 114) were multimodal in some traits due to slight differences in conditions among different microscopes (*i.e.*, confounding factors) of the same composition. The effects of these confounding factors were remarkable in morphological traits related to image intensity. The distributions of these traits were different between microscopes and similar within each microscope in particular periods during image acquisition, but were different even within the same microscope between periods corresponding to the replacement of microscope parts (*e.g.*, filter set, mercury-arc valve). Thus, we defined the confounding factor as a combination of the microscope used and the timing of image acquisition. To minimize the effects of confounding factors, we introduced the GLM by constructing a linear model (one-way analysis of variance) of the confounding factors (CFM: confounding factor model) as formulated below:

$$f\left( y \right)=\beta_{1}MS1+\beta_{2}MS2+\beta_{3}MS2a+\beta_{4}MS2b+\beta_{5}MS3$$

where *y*, *MS1*, *MS2*, *MS2a*, *MS2b*, *MS3*, *β*, and *f* indicate fitted value, microscope 1, microscope 2 before replacement of parts, microscope 2 after parts used over time, microscope 2 after replacement of the microscope parts, microscope 3, fixed effect of each confounding factor, and link functions, respectively. The link function *f* was defined as follows: *f*(*y*) = log(*y*) for *GA*, *f*(*y*) = log[*y* / (1 - *y*)] for *BE* and *BB*, and *f*(*y*) = *y* for *NO*.

To identify morphological traits affected by the confounding factors, we compared AIC between CFM and the null model (NLM) after the MLE. In 337 of the 501 traits, CFM was selected because it had the lowest AIC among the tested models (S1 Table), indicating that more than 60% of the morphological traits were affected by the confounding factors. In this process, BB and BI were selected for 18 and 43 of the 61 traits, respectively, from one of four combinations between PDFs (BB or BI) and the linear models (CFM or NLM) as listed in S1B Table.

## Z transformation of morphological data by Wald test

Once the MLE reached convergence, we transformed the morphological data of heterozygotes to Z values by Wald test. The Z value of Wald test in the *i*th trait of the *j*th gene of the mutants was calculated by the following function:

$$Z_{ij}= \frac{\left( \beta_{ij}-\beta_{i0} \right)}{{SE}_{ij}}$$

where *β_i0_*, *β_ij_*, and *SE_ij_* are the MLE for 114 replicates of wild type, MLE of the *j*th mutant, and standard error. Z values were calculated by summary.gamlss of R function [6]. In traits of the beta-binomial, the Z value was set to zero if the numerator of mutants equaled the number of the estimated value from wild type. In the other cases, the Z value was set to the maximum value among the other mutants.

# References

1. Levy SF, Siegal ML. Network Hubs Buffer Environmental Variation in *Saccharomyces cerevisiae*. Plos Biology. 2008;6(11):2588-604. <https://doi.org/10.1371/journal.pbio.0060264> PMID: 18986213.

2. Yvert G, Ohnuki S, Nogami S, Imanaga Y, Fehrmann S, Schacherer J, et al. Single-cell phenomics reveals intra-species variation of phenotypic noise in yeast. BMC Syst Biol. 2013;7:54. <https://doi.org/10.1186/1752-0509-7-54> PMID: 23822767.

3. Wang K, Wang M, Tang D, Shen Y, Miao C, Hu Q, et al. The role of rice HEI10 in the formation of meiotic crossovers. PLoS Genet. 2012;8(7):e1002809. <https://doi.org/10.1371/journal.pgen.1002809> PMID: 22792078.

4. Taniguchi Y, Choi PJ, Li GW, Chen H, Babu M, Hearn J, et al. Quantifying E. coli proteome and transcriptome with single-molecule sensitivity in single cells. Science. 2010;329(5991):533-8. <https://doi.org/10.1126/science.1188308> PMID: 20671182.

5. Zeyl C, DeVisser JA. Estimates of the rate and distribution of fitness effects of spontaneous mutation in *Saccharomyces cerevisiae*. Genetics. 2001;157(1):53-61. PMID: 11139491.

6. Stasinopoulos DM, Rigby RA. Generalized additive models for location scale and shape (GAMLSS) in R. J Stat Softw. 2007;23(7). <https://doi.org/10.18637/jss.v023.i07>.

7. Octavio LM, Gedeon K, Maheshri N. Epigenetic and conventional regulation is distributed among activators of *FLO11* allowing tuning of population-level heterogeneity in its expression. PLoS Genet. 2009;5(10):e1000673. <https://doi.org/10.1371/journal.pgen.1000673> PMID: 19798446.

8. Bertl A, Slayman CL, Gradmann D. Gating and conductance in an outward-rectifying K^+^ channel from the plasma membrane of *Saccharomyces cerevisiae*. J Membr Biol. 1993;132(3):183-99. <https://doi.org/10.1007/BF00235737> PMID: 8492306.

9. Koppes LJ, Grover NB. Relationship between size of parent at cell division and relative size of its progeny in *Escherichia coli*. Arch Microbiol. 1992;157(5):402-5. PMID: 1510565.

10. Kannaste O, Suomi T, Salmi J, Uusipaikka E, Nevalainen O, Corthals GL. Cross-correlation of spectral count ranking to validate quantitative proteome measurements. Journal of proteome research. 2014;13(4):1957-68. <https://doi.org/10.1021/pr401096z> PMID: 24611565.

11. Kariv G, Paul M, Shani V, Muchtar E, Leibovici L. Benchmarking inappropriate empirical antibiotic treatment. Clinical microbiology and infection : the official publication of the European Society of Clinical Microbiology and Infectious Diseases. 2013;19(7):629-33. <https://doi.org/10.1111/j.1469-0691.2012.03965.x> PMID: 22805537.

12. Pham TV, Piersma SR, Warmoes M, Jimenez CR. On the beta-binomial model for analysis of spectral count data in label-free tandem mass spectrometry-based proteomics. Bioinformatics. 2010;26(3):363-9. <https://doi.org/10.1093/bioinformatics/btp677> PMID: 20007255.
