# Supplementary figures and images for: High-dimensional single-cell phenotyping reveals extensive haploinsufficiency
Source: PLoS Biol. 2018 May 16;16(5):e2005130. doi: 10.1371/journal.pbio.2005130 (PMC5955526; doi:10.1371/journal.pbio.2005130)

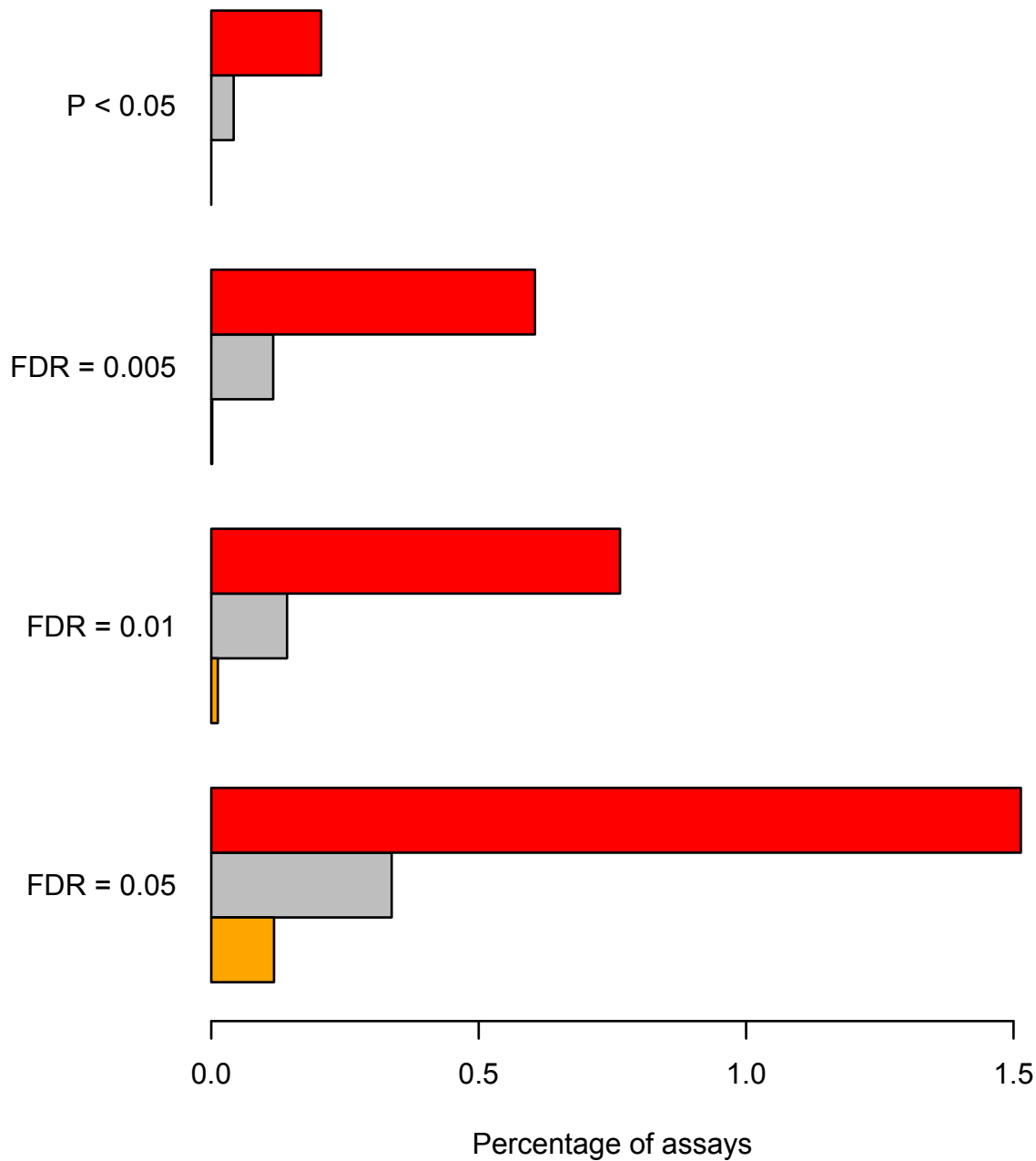

Supplement: S1 Fig — Red, gray, and orange bars indicate frequencies of observed haploinsufficiency phenotypes in essential genes (n = 557,112), nonessential genes (n = 50,100), and wild type (n = 57,114), respectively (S1 Data). Family-wise error rate of P < 0.05 was estimated by Bonferroni correction (n = 557,112). (PDF) [file pbio.2005130.s010.pdf]

A

FDR = 0.05

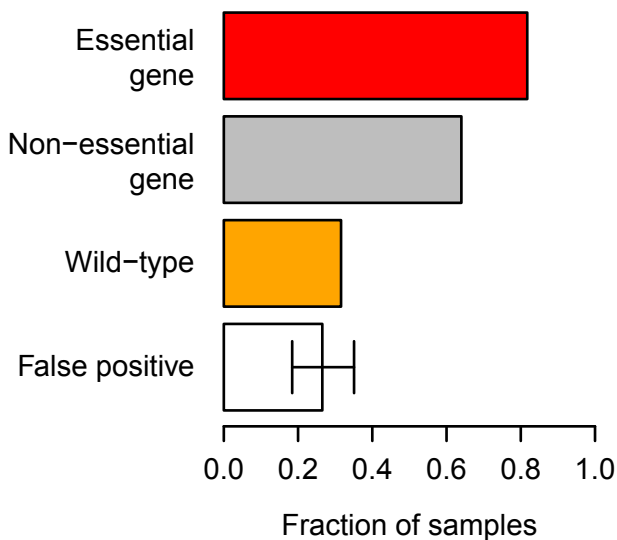

B

FDR = 0.01

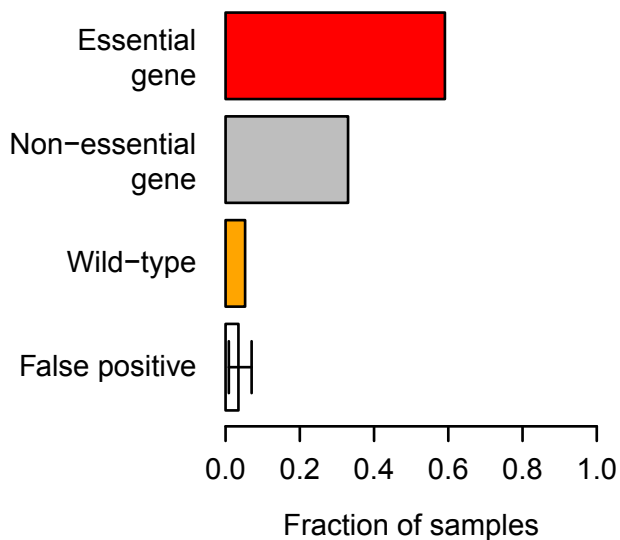

C

FDR = 0.005

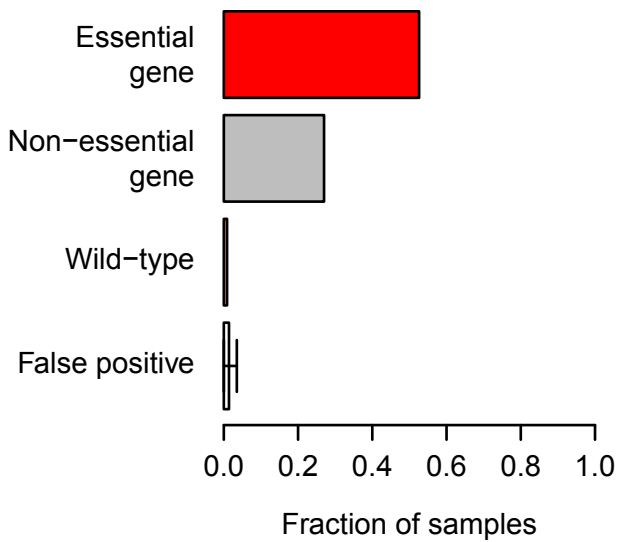

D

P &lt; 0.05

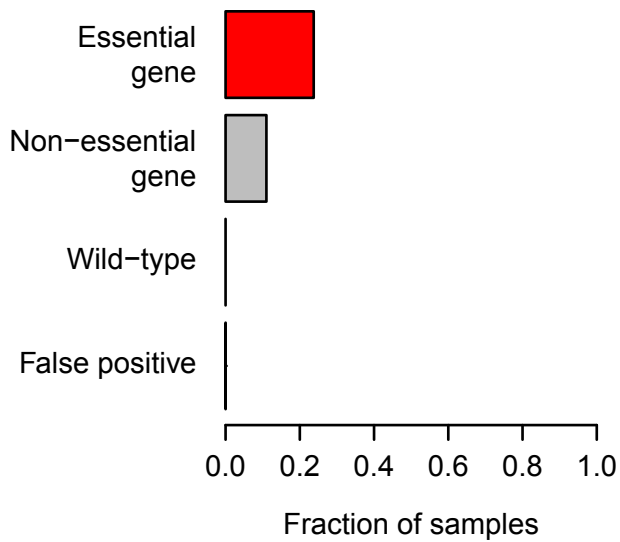

Supplement: S2 Fig — Fraction of the samples in which at least 1 trait was detected at (A) FDR = 0.05 (P < 7.57 × 10^−4^), (B) FDR = 0.01 (P < 7.64 × 10^−5^), (C) FDR = 0.005 (P < 3.02 × 10^−5^), and (D) P < 0.05 after Bonferroni correction (P < 0.05/557,112) (S1 and S4 Data). False positive indicates the percentage of samples detected by chance, which was estimated for wild type using parametric bootstrap resampling. Error bars indicate 95% CIs. FDR, false discovery rate. (PDF) [file pbio.2005130.s011.pdf]

A

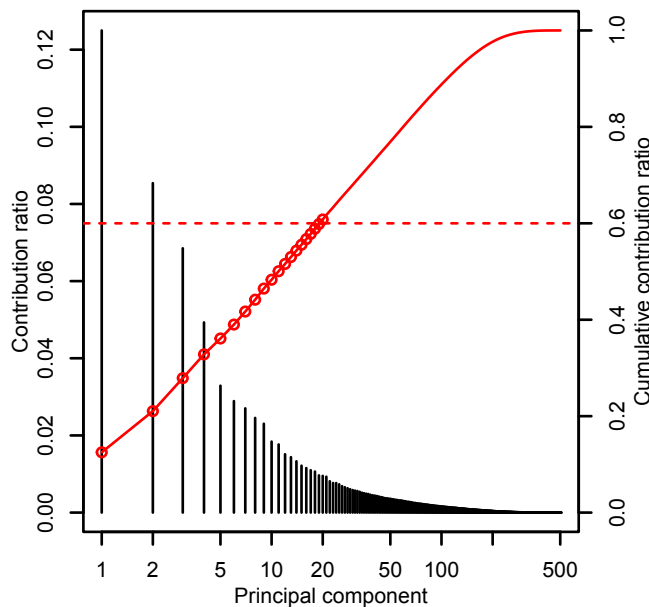

B

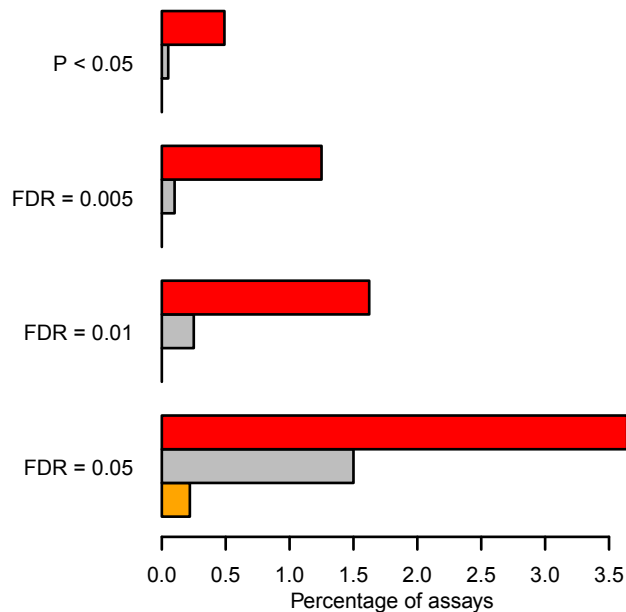

C

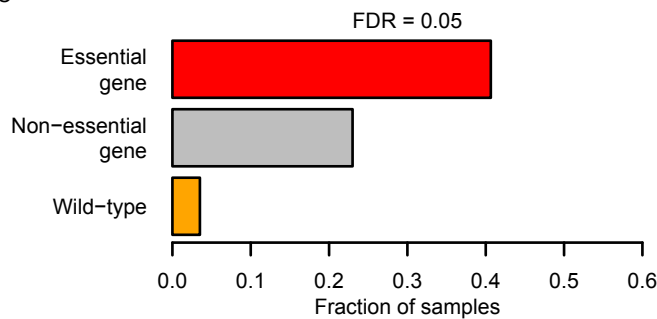

D

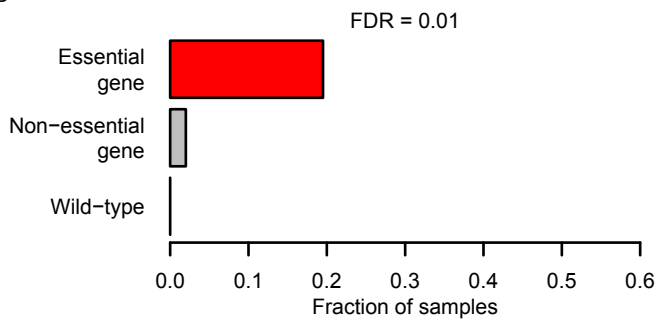

E

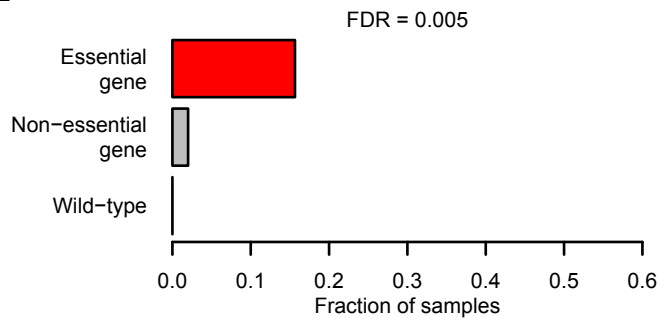

F

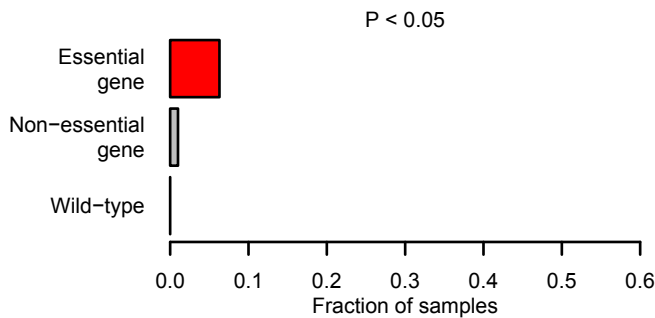

Supplement: S3 Fig — (A) CCR (S5 Data). The black bars (left axis) indicate the contribution ratio, the red circles (right axis) indicate the CCR, and the horizontal dashed lines (right axis) indicate 60% of the CCR. The first 20 PCs that covered 60% of variance were used for detection of the haploinsufficiency phenotype. (B) Percentage of detected phenotypes in all tested assays (S5 Data). Red, gray, and orange boxes indicate essential genes (22,240 assays), nonessential genes (2,000 assays), and wild type (2,280 assays), respectively. Number of heterozygotes detected in at least 1 PC at (C) FDR = 0.05 (P < 2.38 × 10^−3^), (D) FDR = 0.01 (P < 2.12 × 10^−4^), (E) FDR = 0.005 (P < 8.19 × 10^−5^), and (F) P < 0.05 after Bonferroni correction (P < 0.05/22,240) by 1-sample 2-tailed test with Gaussian distribution (S5 Data). CCR, cumulative contribution ratio; PC, principal component; PCA, principal component analysis. (PDF) [file pbio.2005130.s012.pdf]

A

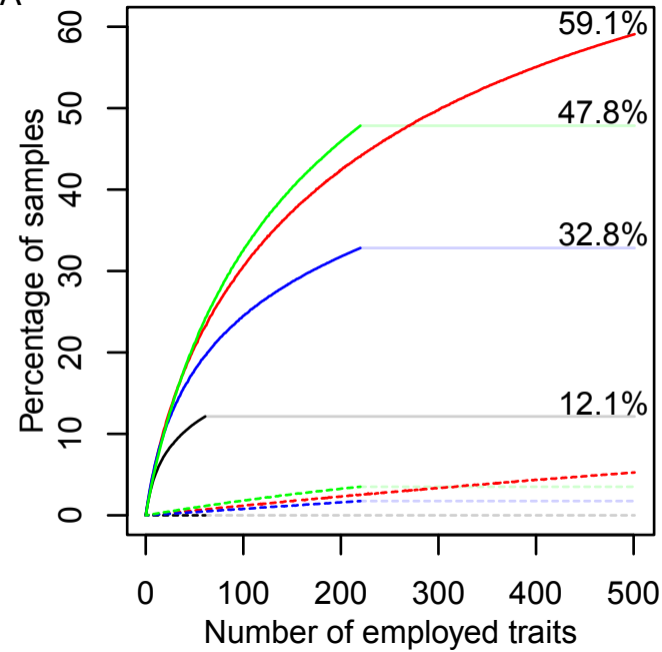

B

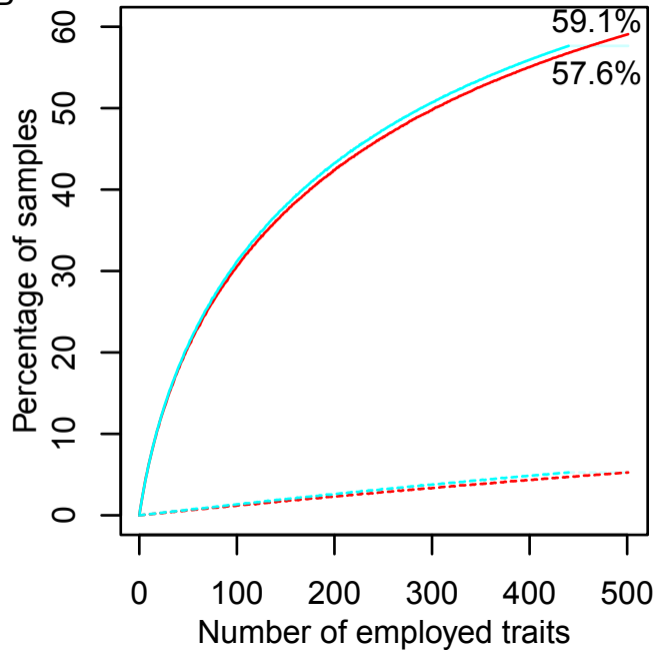

Supplement: S4 Fig — (A) Comparison among mean, noise, and ratio traits (S6 Data). The percentage of heterozygotes (solid line) and wild type (dashed line) detected in at least 1 trait in 1,112 essential genes were compared among 220 noise traits (green), 220 mean traits (blue), 61 ratio traits (black), and all 501 traits (red). Horizontal lines indicate the maximum percentage with each type of trait. (B) Contribution of “noise + mean” traits (S6 Data). Cyan lines indicate 440 “noise + mean” traits. The other symbols are the same as in panel A. (PDF) [file pbio.2005130.s013.pdf]

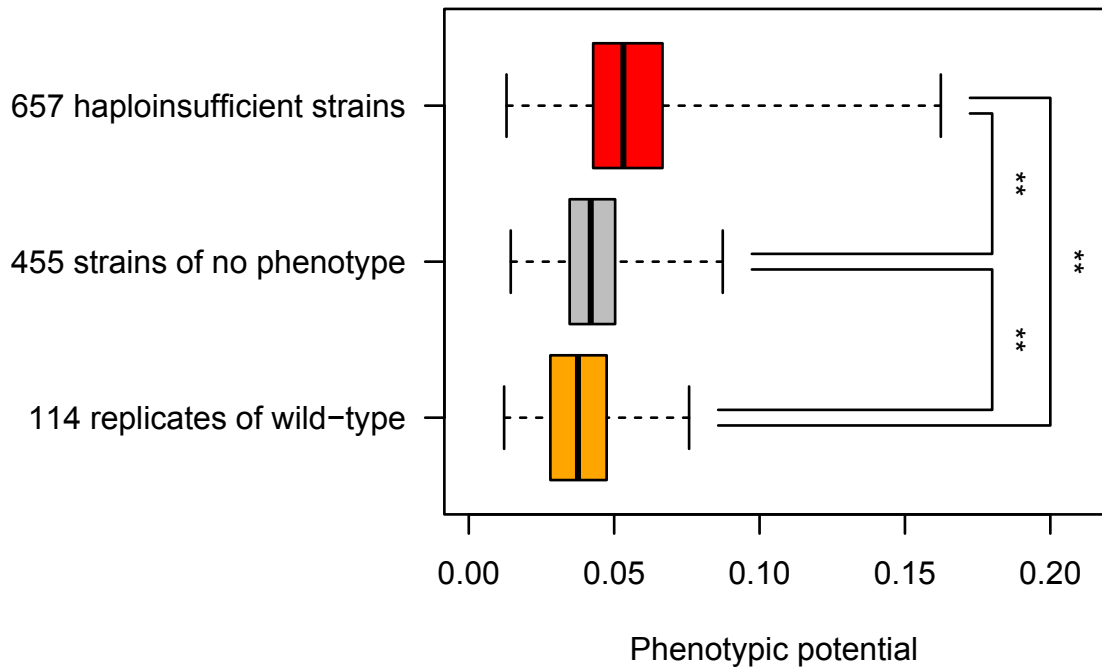

Supplement: S5 Fig — The phenotypic variance in morphology was calculated in terms of the phenotypic potential (x-axis) (S7 Data), as described previously [37]. Asterisks indicate that applying Bonferroni correction to the Mann–Whitney U test yielded P < 0.01. (PDF) [file pbio.2005130.s014.pdf]

444 traits

830 GOs

130 pPCs

346 gPCs

21 21  
pCVs gCVs

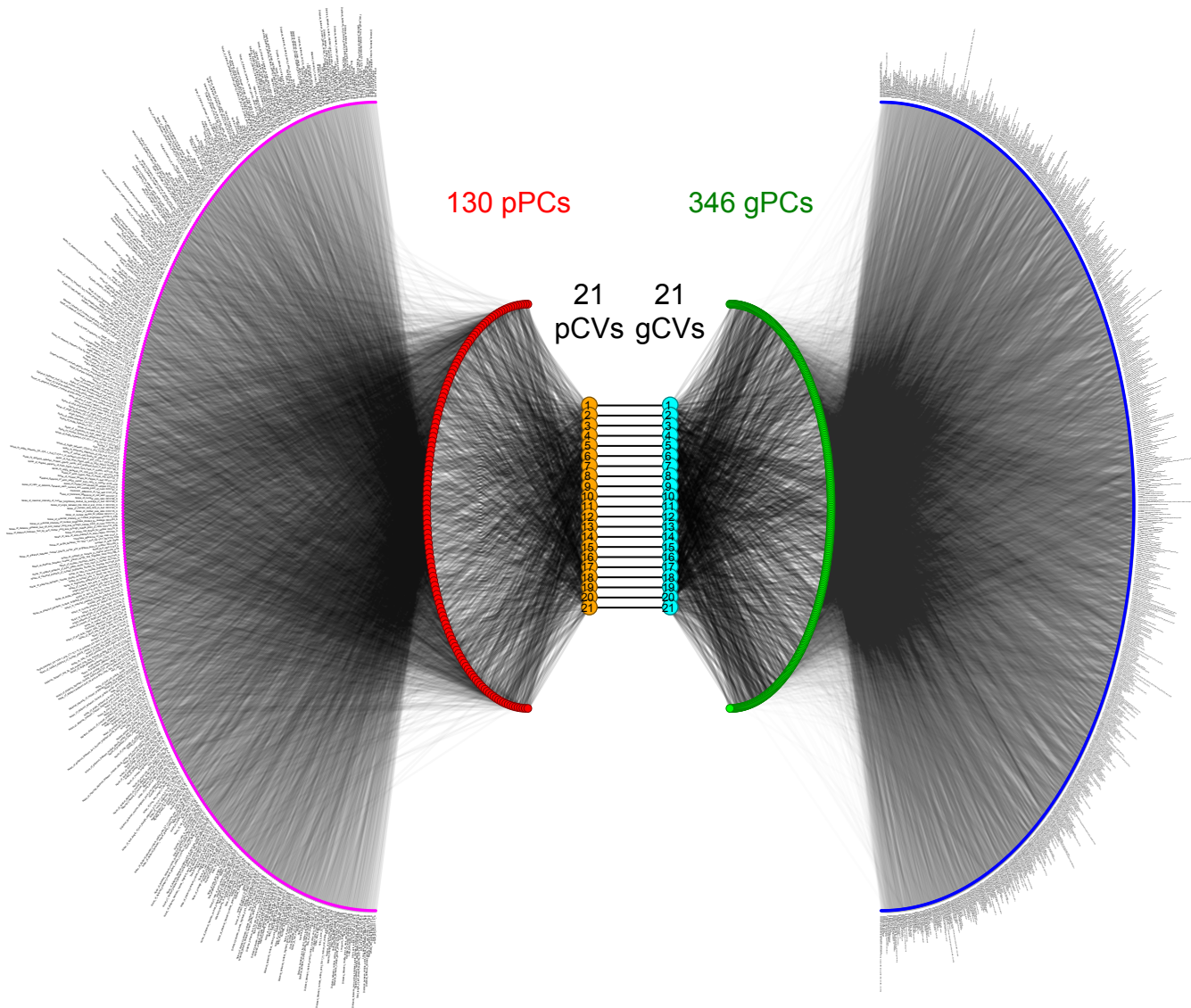

Supplement: S6 Fig — The eye diagram [51] illustrates the CCA procedure. Magenta, red, orange, cyan, green, and blue circles indicate 444 traits, 130 pPCs, 21 pCVs, 21 gCVs, 346 gPCs, and 830 GO terms, respectively. Edges were drawn to have high loadings by cutting with threshold at P < 0.05 by t test for the loading such that each node has more than 1 relationship to other nodes. CCA, canonical correlation analysis; CV, canonical variable. (PDF) [file pbio.2005130.s015.pdf]

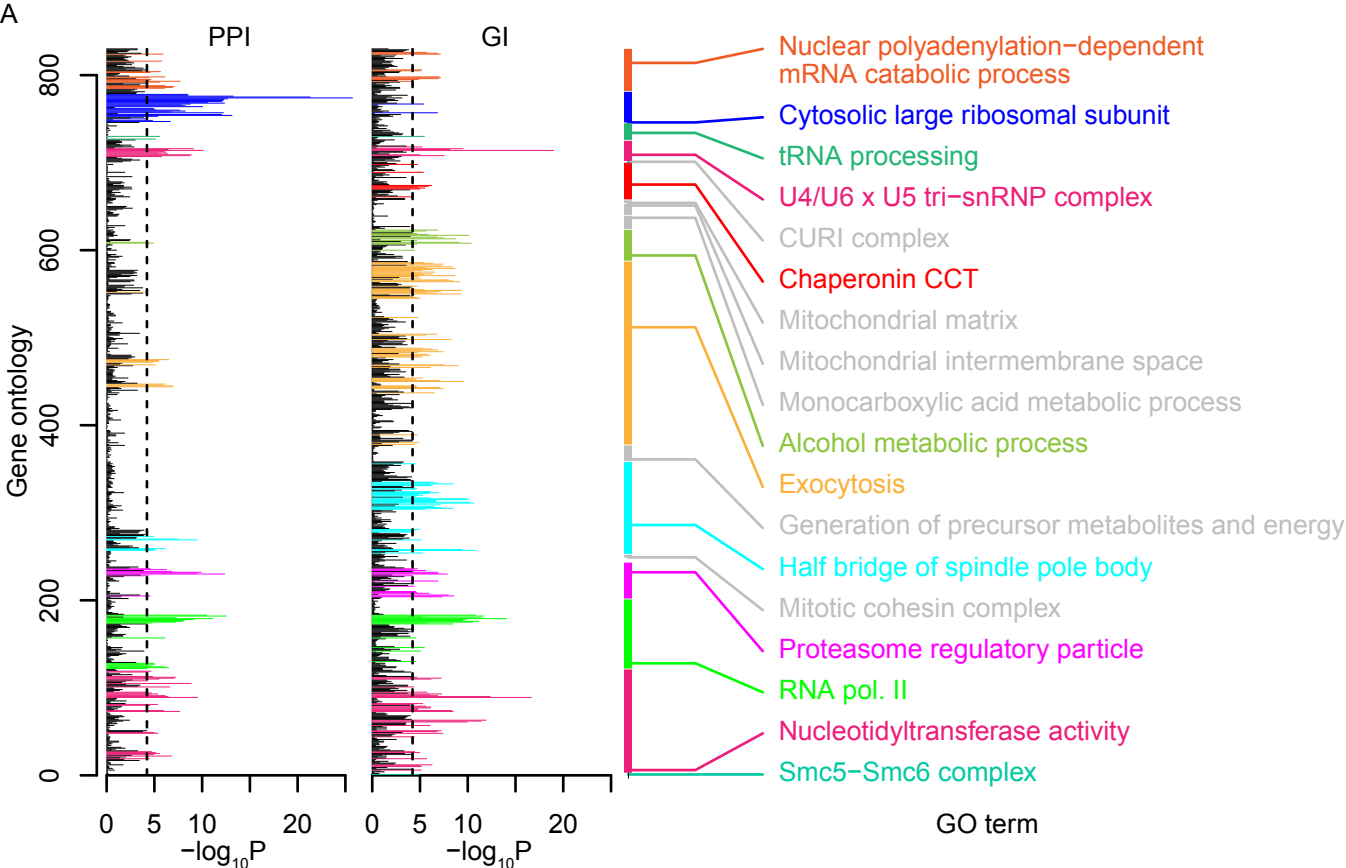

**B**

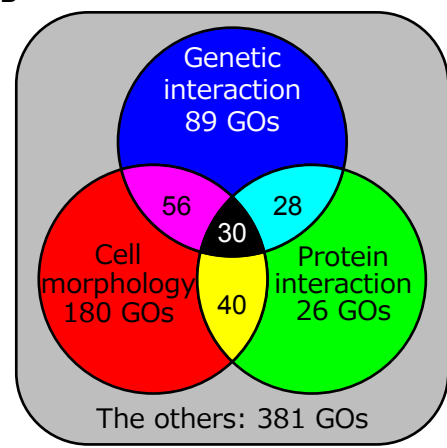

Supplement: S7 Fig — (A) Detection of enriched genes in 2 datasets. In each GO, logistic regression of the interaction degree was applied to the GO annotation of 1,044 genes for PPI [52] and 940 genes for GI [26]. Enrichment of the genes annotated to each GO with a high degree of interaction was assessed using a 1-tailed Wald test for the slope of the linear model at P < 0.05 after Bonferroni correction. Color peaks indicate P values of the 1-tailed Wald test for each of 830 GOs. The vertical dashed line indicates P < 0.05 after Bonferroni correction. Colors of peaks and text for 124 and 201 GOs in PPI and GI, respectively, indicate the GO group and representative GO term, which are the same as in Fig 3. Black peaks and grey texts indicate no correlation was detected at P < 0.05 after Bonferroni correction. (B) Venn diagram of the enriched GO terms in the 3 datasets. The GOs detected in each dataset (306 GOs for cell morphology shown in Fig 3, 124 GOs for protein interaction [52], and 201 GOs for genetic interaction [26]) were summarized in a Venn diagram. GI, genetic interaction; PPI, protein–protein interaction. (PDF) [file pbio.2005130.s016.pdf]

A

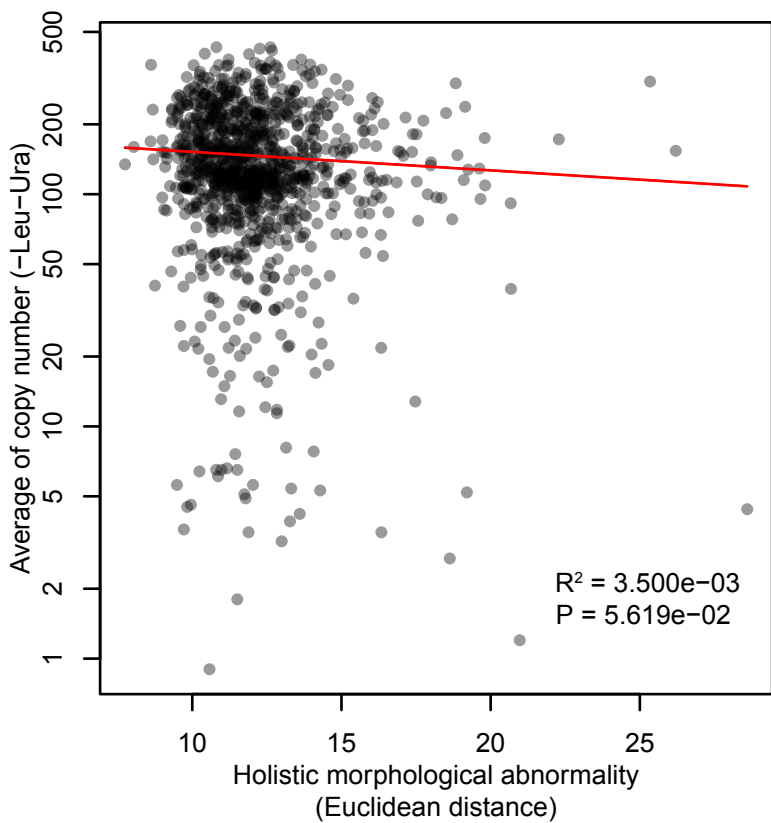

B

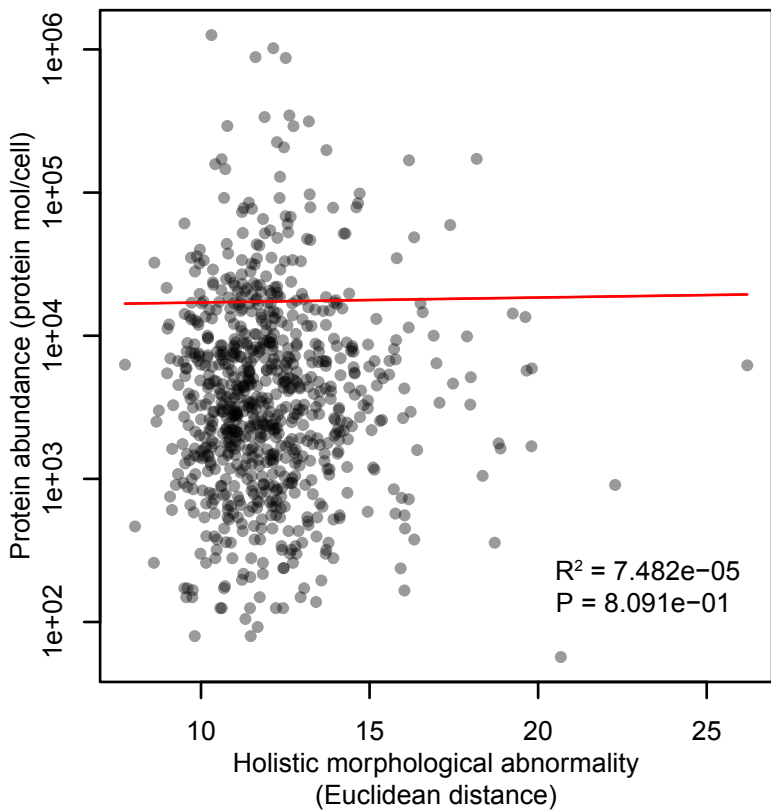

Supplement: S8 Fig — (A) Distribution of copy number limit and morphological abnormality. Gray circles indicate 1,040 essential genes available in both datasets. y- and x-Axes indicate the average copy number limit [20] and morphological abnormality, which was calculated as the Euclidean distance from the mean of the wild type to each heterozygote to obtain a Z value, as described previously [53]. (B) Distribution of protein abundance and morphological abnormality. Gray circles indicate 780 essential genes available in both datasets. y- and x-Axes indicate protein abundance in the cell [21] and morphological abnormality, respectively, which is the same as in panel A. Red line indicates linear regression with a gamma distribution. R2 indicates coefficient of determination. P values were estimated by likelihood ratio test. (PDF) [file pbio.2005130.s017.pdf]

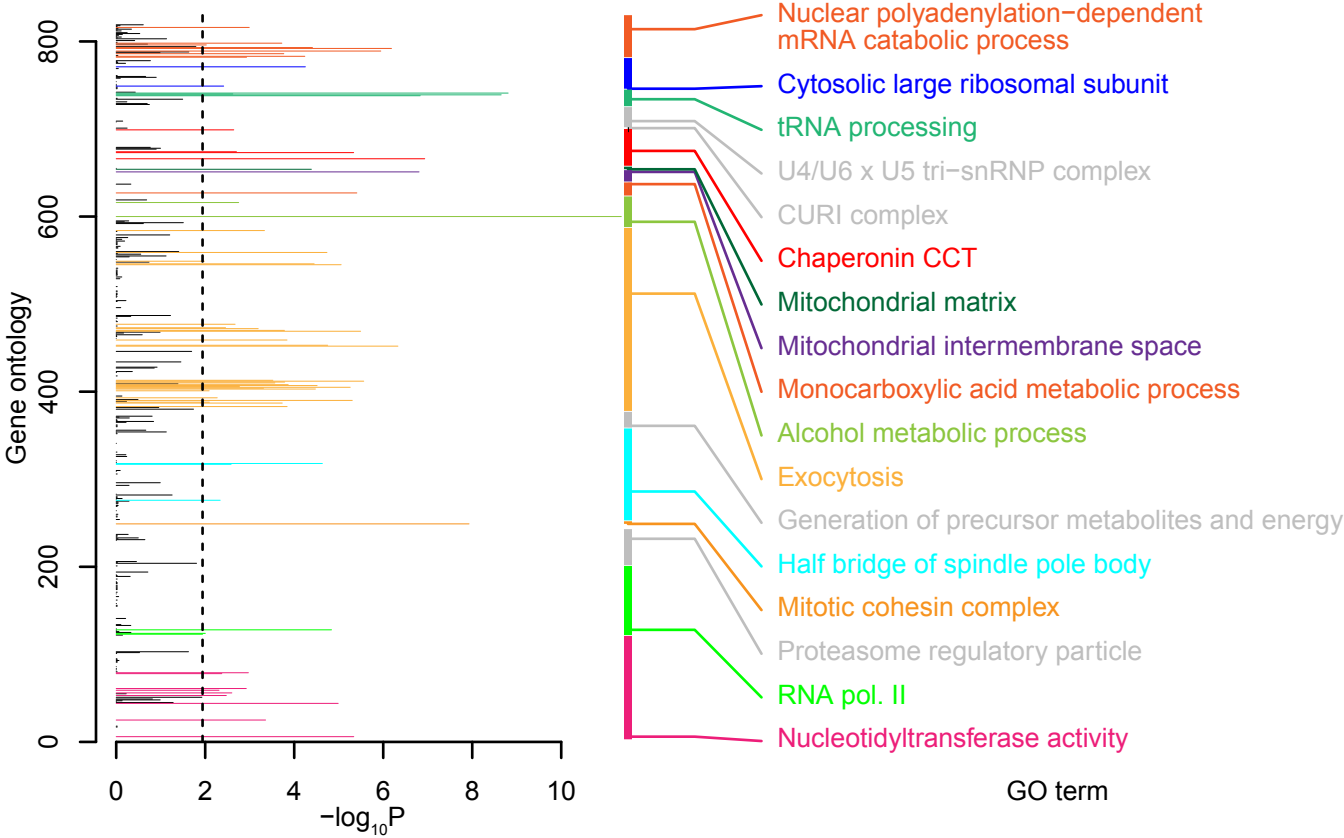

Supplement: S9 Fig — Correlation between protein abundance and function-specific morphological phenotype were assessed using linear regression with a gamma distribution. Function-specific morphological phenotypes were identified using the best combination of pCVs for each GO term, as described in Fig 3. Color peaks indicate P values of 1-tailed Wald test for the slope of the linear model in each of the 306 GOs described in Fig 3. The vertical dashed line indicates FDR = 0.05. Colors of peaks and text indicate GO group and representative GO term, which are the same as in Fig 3. Black peaks and grey texts indicate that no correlation was detected at FDR = 0.05. (PDF) [file pbio.2005130.s018.pdf]

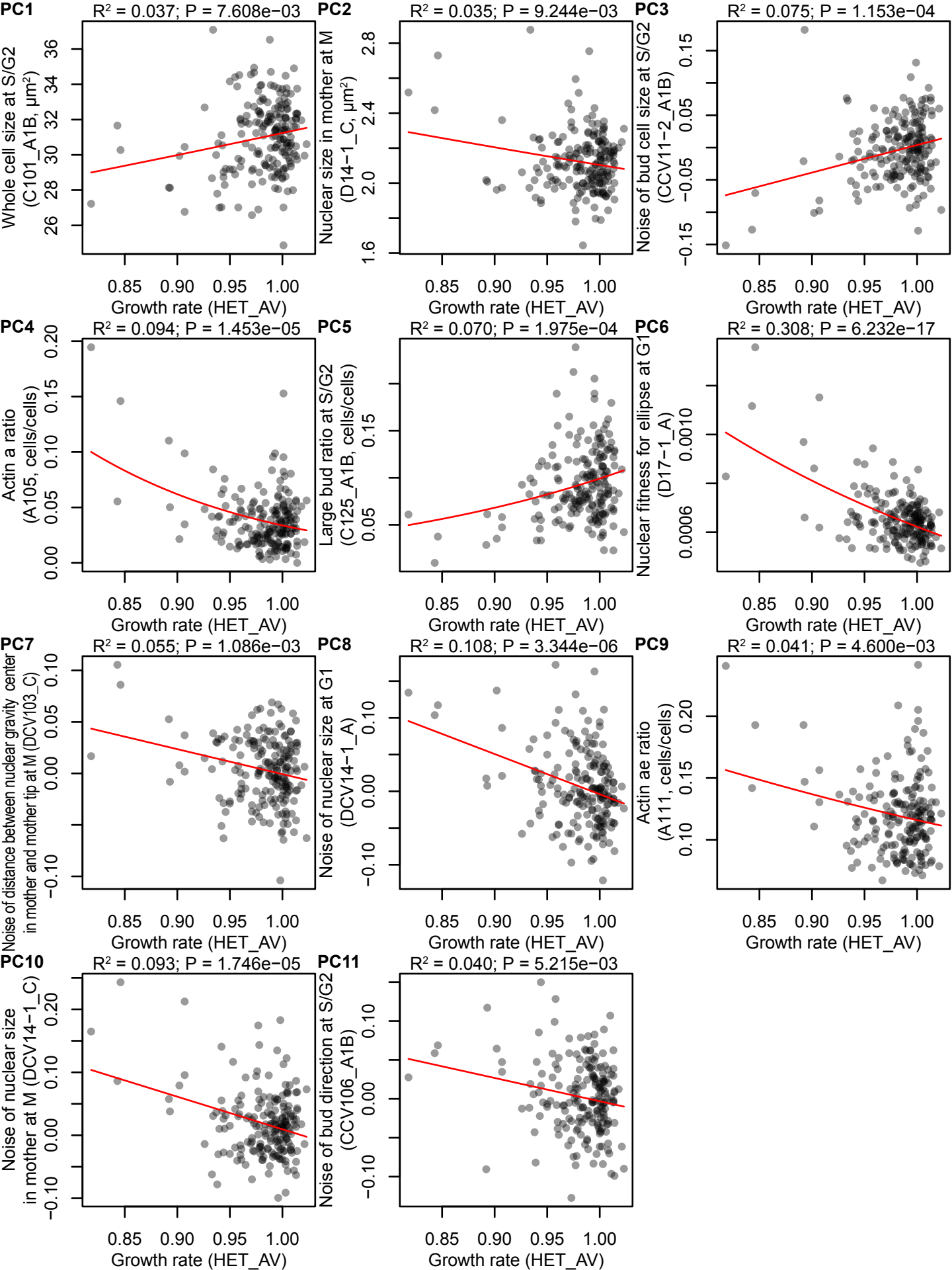

Supplement: S10 Fig — Each independent morphological feature, as defined by PCs (S7 Table), was represented by the morphological traits with significant PC loading at P < 0.05 after Bonferroni correction (t test). The 11 PCs reached 60% of CCR. Red lines indicate linear regressions with the PDFs defined in S1 Table. Legends are the same as in Fig 4. R2 indicates coefficient of determination. P values were estimated by likelihood ratio test. (PDF) [file pbio.2005130.s019.pdf]

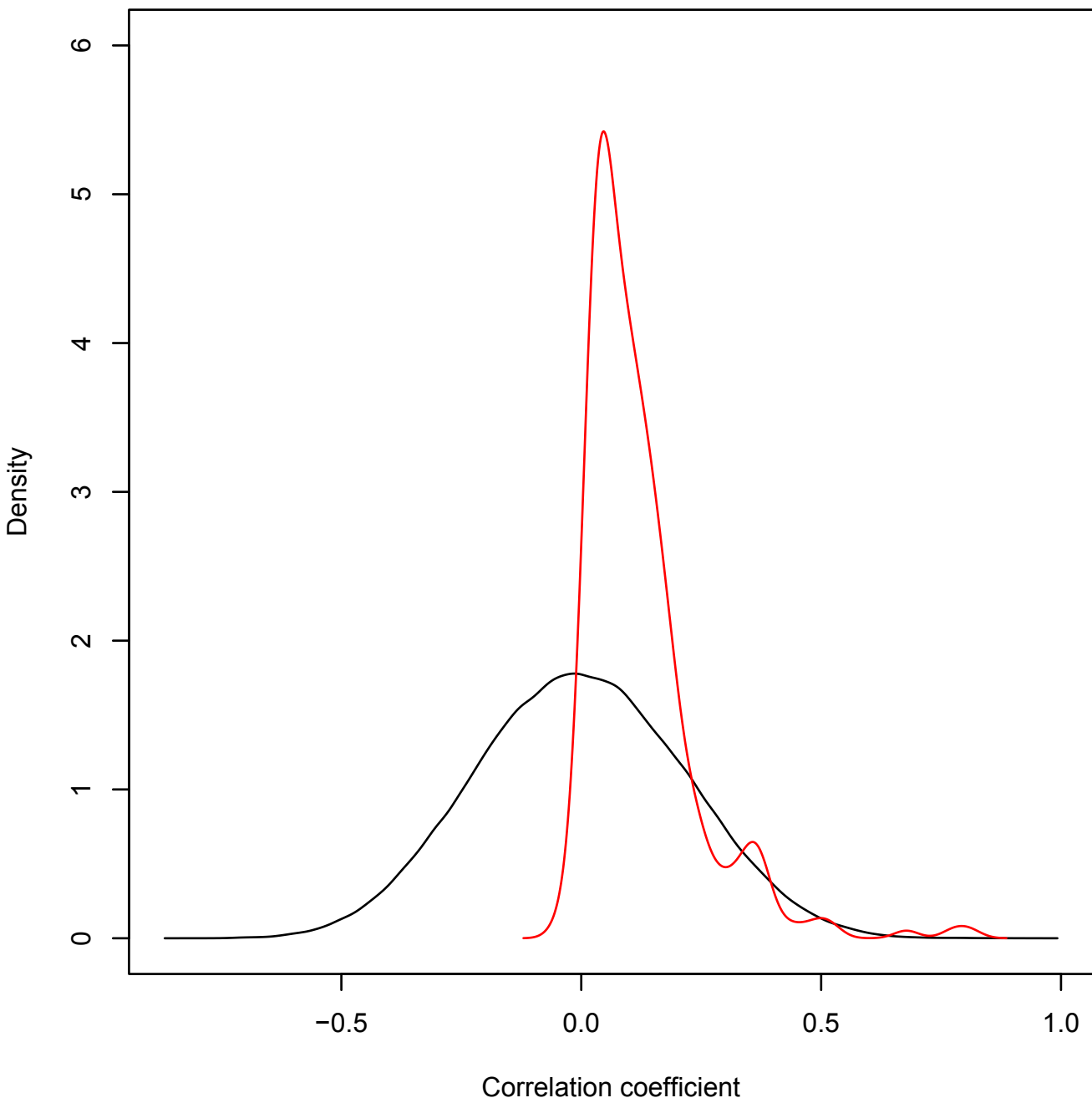

Supplement: S11 Fig — The black curve indicates the distribution of morphology correlation coefficients between pairs of 610 haploinsufficient mutants. The red curve indicates the distribution of mean values of the morphology correlation coefficient within the same GO terms. (PDF) [file pbio.2005130.s020.pdf]

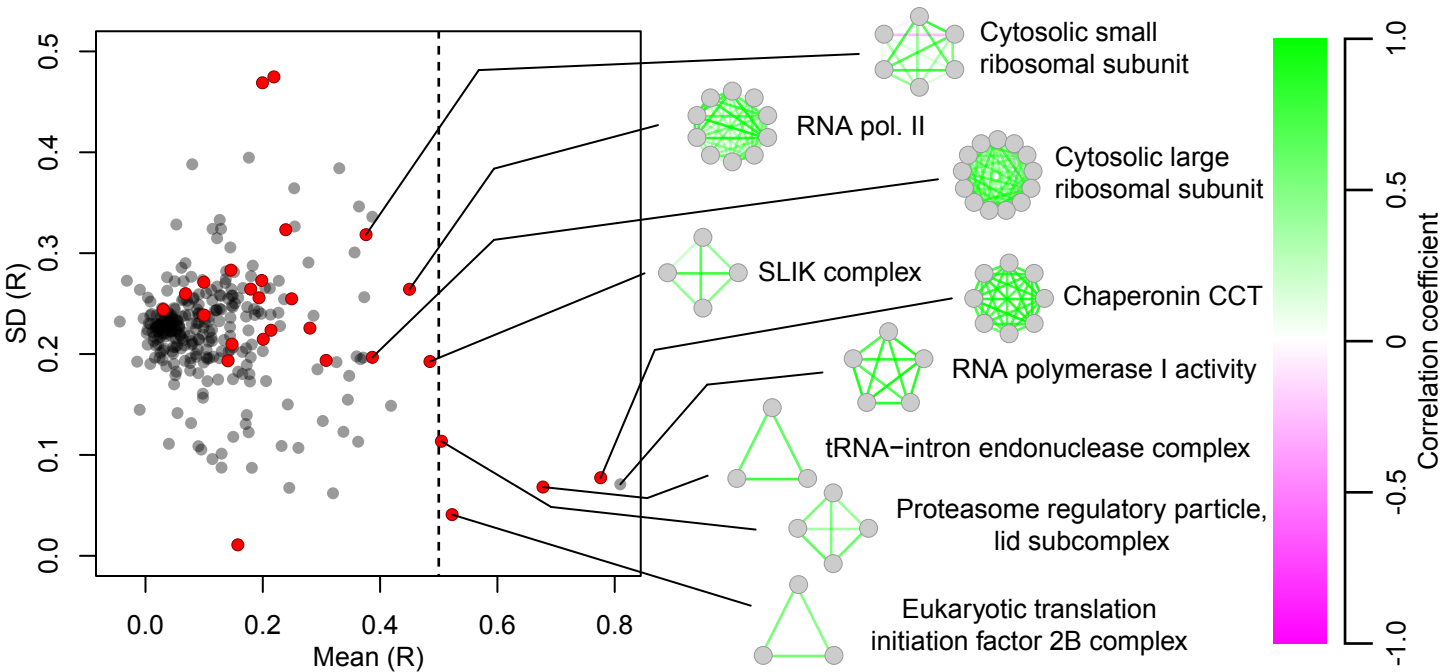

Supplement: S12 Fig — The distribution on the left panel shows the means and SDs of the correlation coefficients of the 306 GOs detected in Fig 3. The red circles indicate GOs that are annotated as protein complexes in the CYC2008 database (http://wodaklab.org/cyc2008/) [50]. The vertical dashed line indicates half of the mean of the correlation coefficient. The network graphs on the right panel show representatives of the GOs. The grey nodes indicate essential genes annotated by the representative GOs: cytosolic small ribosomal subunit (GO:0022627), RNA polymerase II (GO:0005665), cytosolic large ribosomal subunit (GO:0022625), SLIK complex (GO:0046695), chaperonin CCT (GO:0005832), RNA polymerase I activity (GO:0001054), tRNA-intron endonuclease complex (GO:0000214), proteasome regulatory particle, lid subcomplex (GO:0008541), and eukaryotic translation initiation factor 2B complex (GO:0005851). The green and magenta edges in each network indicate positive and negative phenotypic correlations, respectively. GO, gene ontology. (PDF) [file pbio.2005130.s021.pdf]

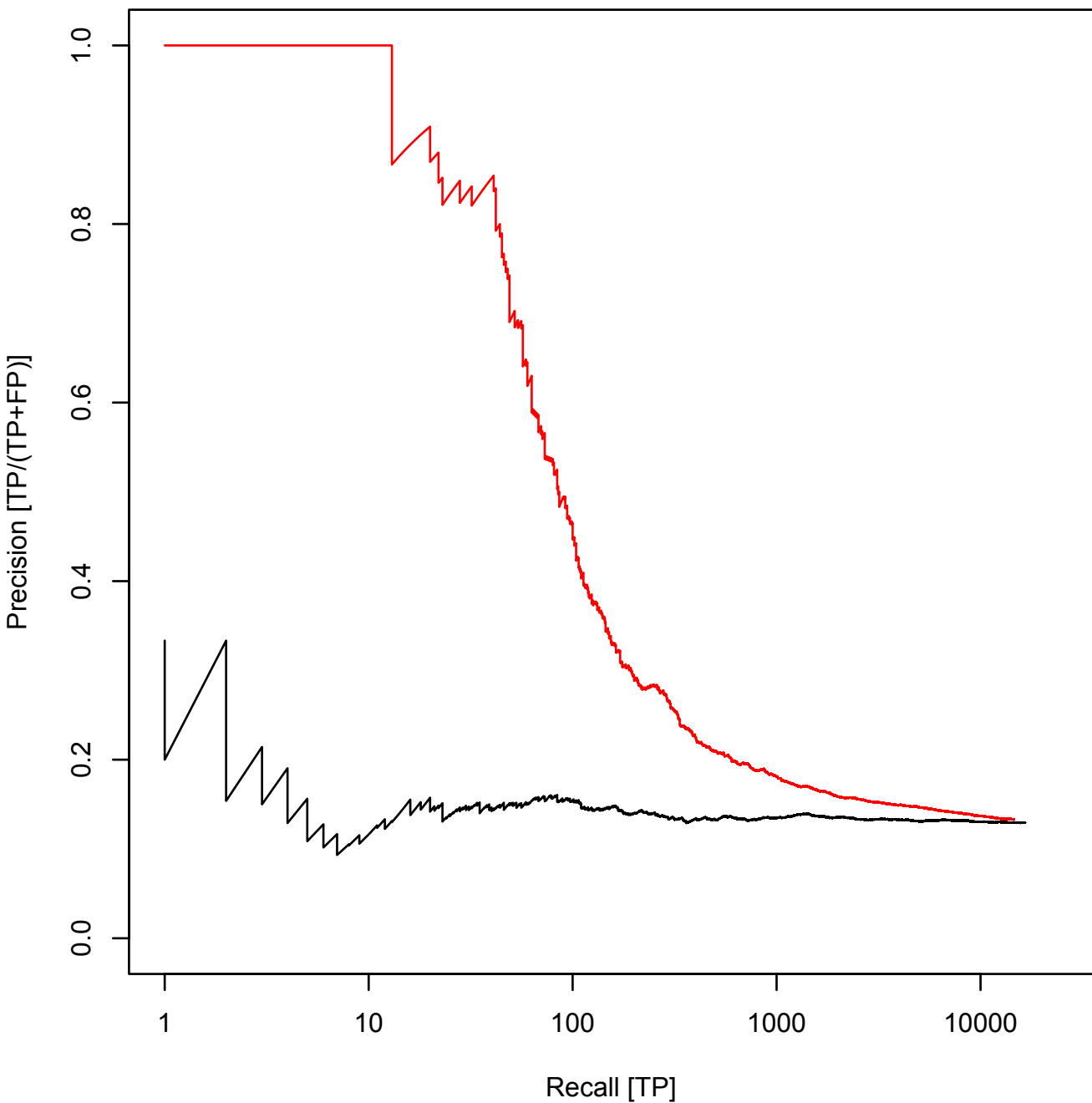

Supplement: S13 Fig — TP and FP indicate the numbers of true positives and false positives, respectively. Black and red lines indicate precision/recall values calculated from pPCs (before CCA) and pCVs (after CCA), respectively. (PDF) [file pbio.2005130.s022.pdf]

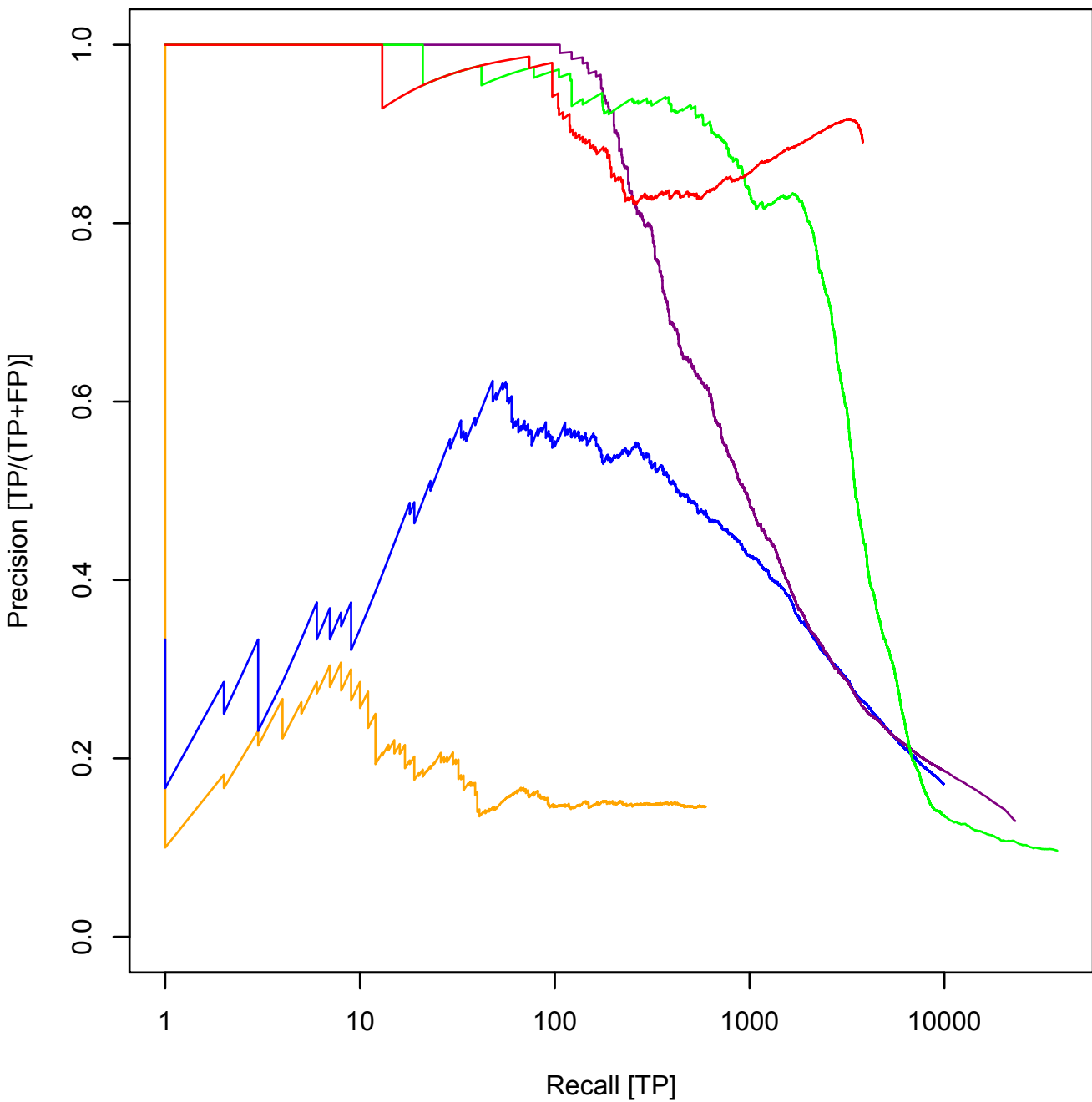

Supplement: S14 Fig — Red: morphological similarity based on haploinsufficient phenotypes (Fig 6A); green: affinity precipitation [23]; purple: similarity of gene expression [24]; blue: synthetic lethality [26]; and orange: phosphorylome network [25]. (PDF) [file pbio.2005130.s023.pdf]
